# Development and assessment of a machine learning tool for predicting emergency admission in Scotland

**DOI:** 10.1038/s41746-024-01250-1

**Published:** 2024-10-23

**Authors:** James Liley, Gergo Bohner, Samuel R. Emerson, Bilal A. Mateen, Katie Borland, David Carr, Scott Heald, Samuel D. Oduro, Jill Ireland, Keith Moffat, Rachel Porteous, Stephen Riddell, Simon Rogers, Ioanna Thoma, Nathan Cunningham, Chris Holmes, Katrina Payne, Sebastian J. Vollmer, Catalina A. Vallejos, Louis J. M. Aslett

**Affiliations:** 1 Department of Mathematical Sciences, Durham University, Durham, UK; 2 Alan Turing Institute, London, UK; 3 MRC Human Genetics Unit, Institute of Genetics and Cancer, University of Edinburgh, Edinburgh, UK; 4 Mathematics Institute, University of Warwick, Coventry, UK; 5 Institute of Health Informatics, University College London, London, UK; 6 Wellcome Trust, London, UK; 7 Public Health Scotland (PHS), Edinburgh, UK; 8 University of St Andrews, St Andrews, UK; 9 NHS National Services Scotland, Edinburgh, UK; 10 Department of Statistics, University of Warwick, Coventry, UK; 11 Department of Statistics, University of Oxford, Oxford, UK; 12 University of Kaiserslautern-Landau, Kaiserslautern, Germany; 13 German Research Centre for Artificial Intelligence, Kaiserslautern, Germany

**Keywords:** Public health, Statistics

## Abstract

Emergency admissions (EA), where a patient requires urgent in-hospital care, are a major challenge for healthcare systems. The development of risk prediction models can partly alleviate this problem by supporting primary care interventions and public health planning. Here, we introduce SPARRA*v*4, a predictive score for EA risk that will be deployed nationwide in Scotland. SPARRA*v*4 was derived using supervised and unsupervised machine-learning methods applied to routinely collected electronic health records from approximately 4.8M Scottish residents (2013-18). We demonstrate improvements in discrimination and calibration with respect to previous scores deployed in Scotland, as well as stability over a 3-year timeframe. Our analysis also provides insights about the epidemiology of EA risk in Scotland, by studying predictive performance across different population sub-groups and reasons for admission, as well as by quantifying the effect of individual input features. Finally, we discuss broader challenges including reproducibility and how to safely update risk prediction models that are already deployed at population level.

## Introduction

**Fig. 1:**
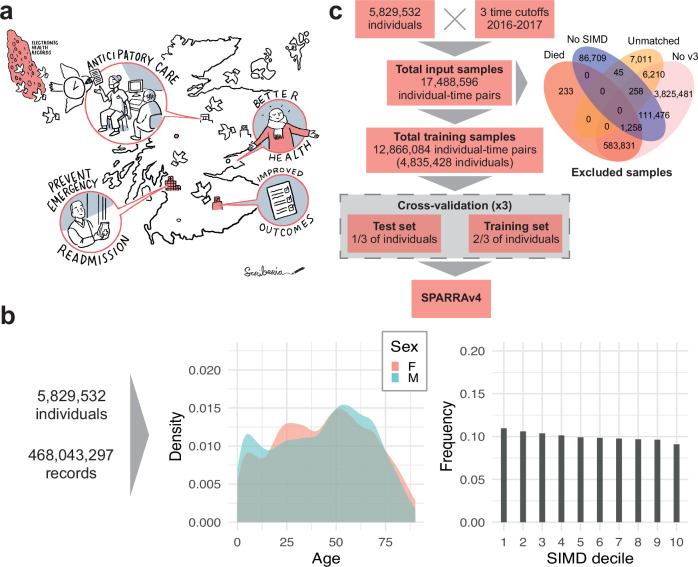
Data and model fitting overview. **a** Illustration of how SPARRA can support primary care intervention with the goal of improving patient outcomes. **b** Distribution of the number of input EHR entries (prior to exclusions) according to age, sex and SIMD deciles (1: most deprived; 10: least deprived). **c** Flow chart summarising data and model fitting pipelines.

Emergency admissions (EA), where a patient requires urgent in-hospital care, represent deteriorations in individual health and are a major challenge for healthcare systems. For example, approximately 395,000 Scottish residents (≈ 1 in 14) had at least one EA between 1 April 2021 and 31 March 2022^1^. In total, around 600,000 EAs were recorded for these individuals, nearly 54% of all hospital admissions in that period, and they resulted in longer hospital stays (6.8 days average) compared to planned elective admissions (3.6 days average). Modern health and social care policies aim to implement proactive strategies^2^, often by appropriate primary care intervention^3–5^. Machine learning (ML) can support such interventions by identifying individuals at risk of EA who may benefit from anticipatory care. If successful, such interventions can be expected to improve patient outcomes and reduced pressures on secondary care (Fig. 1a).

A range of risk prediction models have been developed in this context^6–11^. However, transferability across temporal and geographical settings is limited due to differing demographics and data availability^8^. Development of models in the setting in which they will be used is thus preferable to reapplication of models trained in other settings. In Scotland, the Information Services Division of the National Services Scotland (now incorporated into Public Health Scotland; PHS) developed SPARRA (Scottish Patients At Risk of Re-admission and Admission)—an algorithm to predict the risk of EA in the next 12 months. SPARRA was derived using national electronic health records (EHR) databases and has been in use since 2006. The current version of the algorithm (SPARRA*v*3)^12^ was deployed in 2012/13 and is calculated monthly by PHS for almost the entire Scottish population. Individual-level SPARRA scores can be accessed by general practitioners (GPs), helping them to plan mitigation strategies for individuals with complex care needs. Collectively, SPARRA scores may be used to estimate future demand, supporting planning and resource allocation. SPARRA has also been used extensively in public health research^13–18^.

In this paper we update the SPARRA algorithm to version 4 (SPARRA*v*4) using contemporary supervised and unsupervised ML methods. In particular, we use an ensemble of machine learning methods^19^, and use a topic model^20^ to derive further information from prescriptions and diagnostic data. This represents a large scale ML risk score, fitted and deployed at national level, and widely available in clinical settings. We develop SPARRA*v*4 using EHRs collected for around 4.8 million (after exclusions) Scottish residents between 2013 and 2018. Among other variables, this includes data about past hospital admissions, long term conditions (e.g. asthma) and prescriptions. We use cross-validation to evaluate the validity of SPARRA*v*4 and its stability over time. This shows an improvement of performance with respect to SPARRA*v*3 in terms of discrimination and calibration, including a stratified analysis across different subpopulations. We also perform extensive analyses to determine what reasons for emergency admission are predictable, and use Shapley values^21^ to quantify the effect of individual input factors. Finally, we discuss some of the practical challenges that arise when developing and deploying models of this kind, including issues associated to updating risk scores that are already deployed at population level.

Reproducibility is critical to ensure reliable application of ML in clinical settings^22^. To provide a transparent description of our pipeline, this manuscript conforms to the TRIPOD guidelines^23^ (Supplementary Table 1). Moreover, all code is publicly available at github.com/jamesliley/SPARRAv4. This includes non-disclosive outputs used to generate all the figures and tables presented in this article.

## Results

### Data overview

The input data prior to any exclusions combines multiple national EHR databases held by PHS for 5.8 million Scottish residents between 1 May 2013 and 30 April 2018 (Supplementary Table 2), some of whom died during the observation period. These comprised 468 million records, comprising interactions with the Scottish healthcare system and deaths. The total number of available records varies across sex, age, and SIMD (Fig. 1b), and when records are grouped by database (Supplementary Fig. 1a). In particular, marginally more records are available for individuals in the most deprived areas (as measured by deciles of the 2016 Scottish Index of Multiple Deprivation (SIMD)^24^), particularly within accidents and emergency and mental health hospital records. Two additional tables (see Supplementary Table 2) containing historic data about long term conditions (LTC, back to 1981) and mortality records were also used as input.

We selected three time cutoffs for model fitting (1 May and 1 December 2016, and 1 May 2017) leading to 17.4 million individual-time pairs, hereafter referred to as samples (Fig. 1c). This choice was informed by the extent of data required to define the input features used by the score (3 years prior the time cutoff) and the prediction target (1 year after the time cutoff). We used the earliest (1 May 2016) and latest (1 May 2017) possible time cutoffs, and a third time cutoff halfway between these. Although we could have used more than one time cutoff between the earliest and latest, we deemed that this would add little because, for most patients, we expect to have negiblible variation in their input features and EA status from month to month. After exclusions (which were predominantly due to samples without SPARRA*v*3 scores; see Methods), the data comprise 12.8 million samples corresponding to 4.8 million individuals. Overall, the study cohort is slightly older, has more females, and is moderately more deprived than the general population (Table 1). The prediction target was defined as a recorded EA to a Scottish hospital or death in the year following the time cutoff (see Methods). In total, 1,142,169 EA or death events (9%) were observed across all samples. This includes 57,183 samples for which a death was recorded (without a prior EA within that year) and 1,084,986 samples for which an EA was recorded (amongst those, 107,827 deaths were observed after the EA). As expected, the proportion of deaths amongst the observed events increases with age (Supplementary Fig. 1b). Moreover, patients with an EA or death event (in at least one time cutoff) are, on average, older and more deprived than those without an event (Table 1).

**Table 1:** Demographic summary for the different cohorts: the whole Scottish population (approximately 5.8 million), those present in the input databases at least once (17,488,596 samples comprising 5,829,532 unique individuals), our study cohort after exclusions (12,866,084 samples comprising 4,835,428 unique individuals) and our study cohort after stratifying by event status (EA or death: 1,142,169 samples comprising 667,566 unique individuals; no EA or death: 11,723,915 samples comprising 4,670,756 unique individuals)

	Cohort				
Variable	Scottish population	Input data	After exclusions	EA or death	No EA or death
Sex (%)					
Male	48.5	48.2	45.4	46.2	45.3
Female	51.5	51.8	54.6	53.7	54.7
Age at time cutoff (%)					
0-19	16.9	21.1	19.6	11.8	20.4
20-70	71.2	64.2	64.9	50.1	66.4
71+	11.9	14.7	15.4	38.1	13.2
SIMD decile (%)					
1-5	50.0	50.8	52.0	59.5	51.2
6-10	50.0	49.2	48.0	40.5	48.8
Any LTC (%)	Unknown	29.4	32.1	58.8	29.5

Summary statistics were calculated using sample-level data. The EA or death cohort includes individual-time pairs for which the individual had at least one EA or died during the year after the time. LTC denotes long-term conditions (e.g. epilepsy). Data for the Scottish population is from the 2011 Census^47^.

### Overall predictive performance

**Fig. 2:**
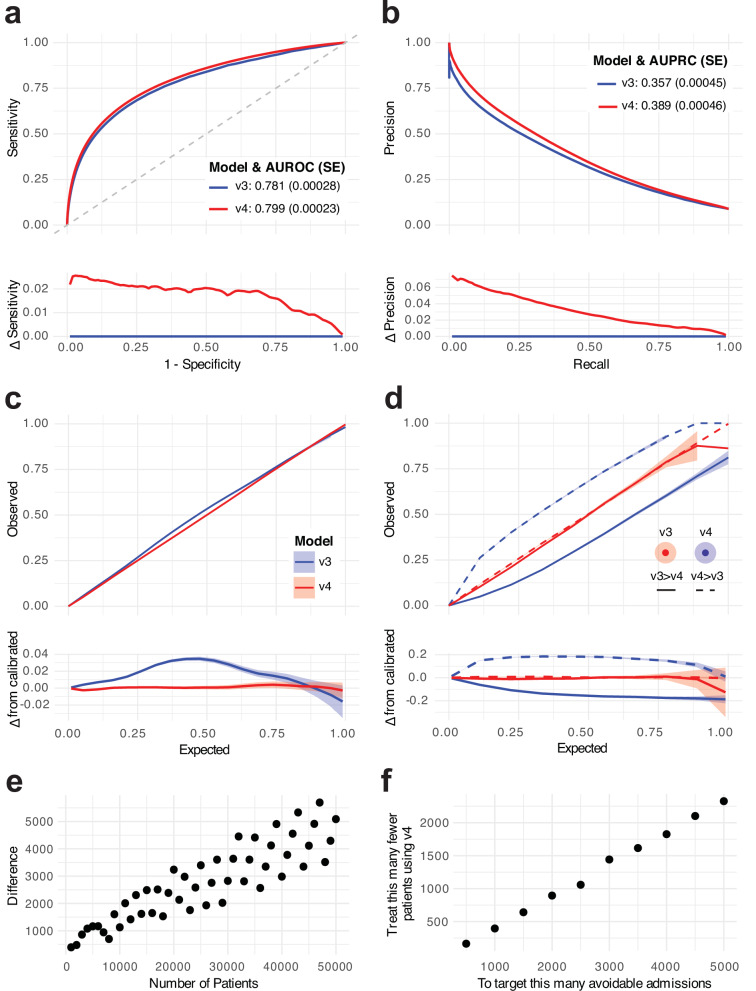
Comparison of overall predictive performance between SPARRA*v*3 and SPARRA*v*4. **a** ROC. **b** PRC. Lower sub-panels show differences in sensitivity and precision, respectively. Confidence intervals are negligible. **c** Calibration curves. **d** Calibration curves for samples in which ∣*v*4 − *v*3∣ > 0.1. Lower sub-panels show the difference between curves and the *y* = *x* line (perfect calibration). Confidence envelopes are pointwise (that is, for each *x*-value, not the whole curve). Predicted/true value pairs are combined across cross-validation folds in all panels for simplicity. **e** Difference in the number of individuals who had an event amongst individuals designated highest-risk by *v*3 and *v*4. The repeating pattern is a rounding effect of *v*3. **f** Difference in the number of highest-risk individuals to target to avoid a given number of admissions.

In held out test data, SPARRA*v*4 was effective at predicting EA, and outperformed SPARRA*v*3 on the basis of area-under-receiver-operator-characteristic curve (AUROC) and area-under-precision-recall-curve (AUPRC) (Fig. 2a, b). SPARRA*v*4 was also better calibrated, particularly for samples with observed risk ≈ 0.5 (Fig. 2c). Whilst SPARRA*v*3 and SPARRA*v*4 scores were highly correlated, large discrepancies were observed for some samples (Supplementary Fig. 2). In samples for whom *v*3 and *v*4 disagreed (defined as ∣*v*3 − *v*4∣ > 0.1), we found that *v*4 was better-calibrated than *v*3 (Fig. 2d).

We also assessed the potential population-wide benefit of SPARRA*v*4 over SPARRA*v*3 directly. Amongst the 50,000 individuals judged to be at highest risk by SPARRA*v*3, around 4000 fewer individuals were eventually admitted that were amongst the 50,000 individuals judged to be at highest risk by SPARRA*v*4 (Fig. 2e). For another perspective, if we simply assume that 20% of admissions are avoidable (value taken from^25^), that avoidable admissions are as predictable as non-avoidable admissions, and that we wish to pre-empt 3000 avoidable admissions by targeted intervention on the highest risk patients (the second assumption is conservative, since avoidable admissions are often predictable due to other medical problems). Then, by using SPARRA*v*4, we would need to intervene on approximately 1,500 fewer patients than if we were to use SPARRA*v*3 in the same way, in order to achieve the target of avoiding 3000 admissions (Fig. 2f).

SPARRA*v*4 comprises an ensemble of models (see Methods), so we also explored a breakdown of AUROC/AUPRC (Table 2) and calibration (Supplementary Fig. 3) across constituent models. The ensemble had slightly better performance (> 1 standard error) than the best constituent models (XGB and RF) and substantially better performance than simple statistical models (GLM and NB), which can be considered as benchmarks. Note that some constituent models (ANN, GLM, NB) had ensemble coefficients which were regularised to be vanishingly small, so in practice scores for those models need not be computed when calculating SPARRA*v*4. We investigated whether performance could be improved by using separate sets of coefficients for each SPARRA*v*3 cohort, but found that the improvement was so small that we judged this to be unnecessary (Supplementary Note 3).

**Table 2:** Overall discrimination performance for SPARRA*v*4 and its constituent models

Model	Fold 1				
	AUROC	AUPRC	Coef.		
ANN	0.7613	0.346	0		
Penalised GLM	0.7879	0.3657	0		
Naive Bayes	0.7471	0.2233	0		
RF, depth: 20	0.7927	0.3787	0.3624		
RF, depth: 40	0.7845	0.3666	0		
SPARRA*v*3	0.7812	0.3568	0		
XGB depth: 4	0.7981	0.3839	0.6626		
XGB depth: 8	0.7984	0.3873	2.004		
XGB depth 3	0.7984	0.3864	1.363		
Ensemble	0.7989	0.3888			

Model	Fold 2				
	AUROC	AUPRC	Coef.		
ANN	0.7698	0.3479	0		
Penalised GLM	0.7874	0.367	0		
Naive Bayes	0.7468	0.2238	0		
RF, depth: 20	0.7928	0.3799	0.3749		
RF, depth: 40	0.7844	0.3678	0		
SPARRA*v*3	0.7809	0.3584	0		
XGB depth: 4	0.7975	0.3839	0.6579		
XGB depth: 8	0.798	0.3881	1.162		
XGB depth 3	0.7981	0.387	1.727		
Ensemble	0.7987	0.3895			

Model	Fold 3			Mean over folds	
	AUROC	AUPRC	Coef.	AUROC	AUPRC
ANN	0.7693	0.3525	0	0.7668	0.3488
Penalised GLM	0.7878	0.3661	0	0.7877	0.3663
Naive Bayes	0.7468	0.2246	0	0.7469	0.2239
RF, depth: 20	0.7926	0.3791	0.5013	0.7927	0.3792
RF, depth: 40	0.784	0.3674	0	0.7843	0.3672
SPARRA*v*3	0.7809	0.3572	0	0.7810	0.3574
XGB depth: 4	0.7973	0.3837	0.9105	0.7976	0.3838
XGB depth: 8	0.7978	0.3877	1.116	0.7981	0.3877
XGB depth 3	0.798	0.3867	1.418	0.7982	0.3867
Ensemble	0.7985	0.3891		0.7987	0.3891

Areas under ROC curves and PR curves by fold for each constituent predictor and ensemble. Columns ‘Coef.’ indicate estimated coefficients (weights) in the final ensemble (see Methods section for details). All standard errors for AUROCs are < 5 × 10^−4^ and for AUPRCs are < 8 × 10^−4^.

### Stratified performance of SPARRA*v*3 and SPARRA*v*4

**Fig. 3:**
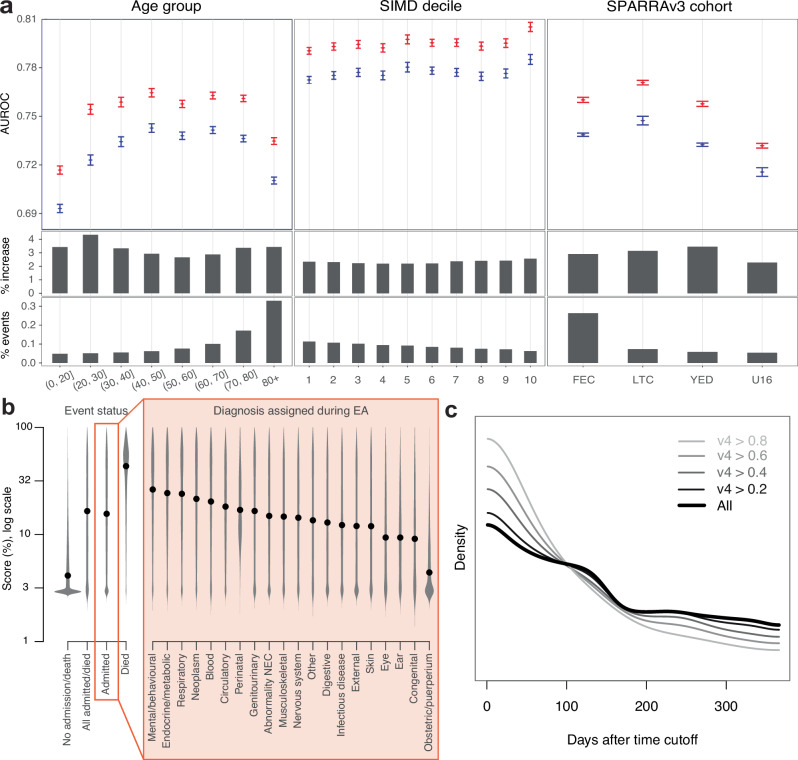
Stratified performance of SPARRA*v*3 and SPARRA*v*4. **a** Performance of SPARRA*v*3 and SPARRA*v*4 in subcohorts defined by age, SIMD and the original subcohorts defined during SPARRA*v*3 development (Methods). Top: AUROC (blue: SPARRA*v*3; red: SPARRA*v*4). Vertical bars denote plus/minus 3 standard deviations. Middle: AUROC increase for SPARRA*v*4 with respect to SPARRA*v*3. For context, bottom sub-panels show the proportion of samples with an event within each group. **b** Distribution of SPARRA*v*4 scores (in log-scale) based on the type of diagnosis recorded during the admission (see Supplementary Table 5 for definitions). Black points indicate the associated medians. Groups were defined according to whether an event was observed (grey violin plots) or, for those with an EA, based on the diagnosis recorded during the admission (black violin plots). **c** Density of time-to-first-EA (that is, days between time cutoff and first EA date) in subsets of individuals who had an EA in the year following the time cutoff and had a SPARRA*v*4 score above a given cutoff. For instance, the lightest line shows density of time-to-first-EA in samples who had an EA and had SPARRA*v*4 > 0.8.

To examine differences in performance more closely, we explored the performance of SPARRA*v*3 and SPARRA*v*4 across different patient subcohorts defined by age, SIMD deciles and the four subcohorts defined as part of SPARRA*v*3 development. Generally, we observed that SPARRA*v*4 had better discrimination performance across all subcohorts (Fig. 3a).

### Conditional performance of SPARRA*v*4 by admission type and imminence

Figure 3 b displays the distribution of SPARRA*v*4 scores stratified according to event status and, for those with an EA, according to the diagnosis that was assigned to the patient during admission (Supplementary Table 5). When comparing samples with and without an event (defined by the composite EA or death outcome), we observed the former had generally lower SPARRA*v*4 scores. Amongst those with an event, all-cause mortality was associated with high SPARRA*v*4 scores. If the event was an EA, we found that samples with certain medical classes of admission tended to have particularly high SPARRA scores, suggesting that such admissions can be predicted disproportionately well (Fig. 3b): in particular, those with mental/behavioural, respiratory and endocrine/metabolic related admissions. As one would expect, we were less able to predict external causes of admissions (e.g., S21: open wound of thorax^26^). Obstetric and puerperium-related admissions were particularly challenging to predict by SPARRA*v*4. When further analysing SPARRA*v*4 scores, we also found that among individuals who had an EA during the 1 year outcome period, those with higher risk scores were likelier to have the first EA near the start of the period (Fig. 3c). We did not use an absolute threshold to determine who is at high risk. Instead, we ranked individuals according to their scores and looked at those in the top part of the ranking (i.e. with the highest risk scores).

### Deployment scenario stability and performance attenuation

We next addressed two crucial aspects pertaining to practical usage of SPARRA*v*4. Firstly, we assess the durability of performance for a model trained once (at the time cutoff 1 May 2014, using a one-year lookback) and employed to generate scores at future times (1 May and 1 December 2015, 1 May and 1 December 2016, 1 May 2017), confirming it does not deteriorate. This is the way in which SPARRA*v*4 will be deployed by PHS, generating new scores each month but without repeated model updating, akin to SPARRA*v*3’s monthly use without update from 2013–2023. Secondly, we demonstrate that it is none-the-less necessary to update scores despite the absence of model updates, since evolving patient covariates lead to the performance attenuation of any point-in-time score.

**Fig. 4:**
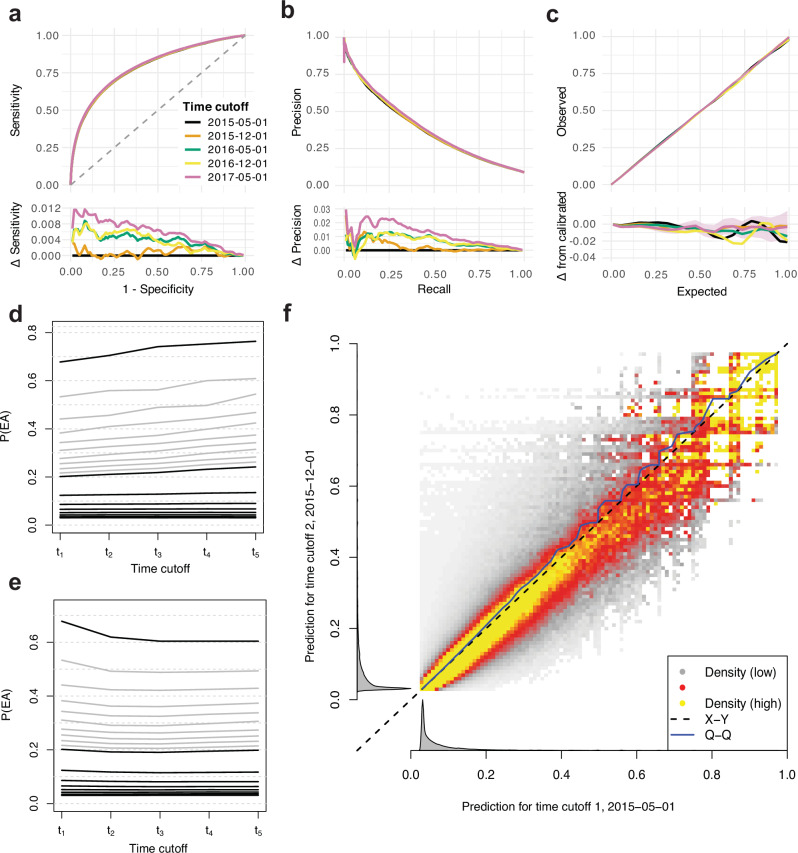
Performance of a static model with changing scores over time. **a**–**c** Performance of scores calculated at *t*_1_ − *t*_5_ from static model *M*_0_. **a** ROC curves. Lower panel shows differences in sensitivity with respect to *t*_1_. **b** PRC curves. Lower panel shows differences in precision with respect to *t*_1_. **c** Calibration curves. Lower panel shows the difference between observed and expected EA frequency. **d** Centiles (grey) and deciles (black) of risk scores (calculated using *M*_0_) over time, across all individuals with data available at all time cutoffs. **e** Average score over time for groups of individuals defined by risk centiles (grey) and deciles (black) at time *t*_0_ (2 May 2015). **f** Density (low to high: white-grey-red-yellow) of scores generated using the static model *M*_0_ to predict EA risk at *t*_1_ (2 May 2015) and *t*_2_ (1 Dec 2015). The density is normalised to uniform marginal on the Y axis, then the X axis; true marginal distributions of risk scores are shown alongside in grey.

We firstly used a *static model* *M*_0_ (Methods) to predict risk at future time-points (i.e. new scores are generated as the features are updated). *M*_0_ performed essentially equally well over time (Fig. 4a–c), with no statistically significant decrease in performance (adjusted p-values > 0.05), or improved performance with time for all comparisons of AUROCs. With stability under the deployment scenario confirmed, we also explored the distribution of scores over time. In line with expectations, the quantiles of scores generated by the static model increased as the cohort grew older (Fig. 4d). The mean risk scores of individuals in the highest centiles of risk at *t*_0_ decreased over time (Fig. 4e), suggesting that very high risk scores tend to be transient. The bivariate densities of time-specific scores (Fig. 4f) also show lower scores to be more stable than higher scores, and that subjects ‘jump’ to higher scores (upper left in Fig. 4f) more than they drop to lower scores (bottom right).

Finally, we examined the behaviour of *static scores* (computed at *t*_0_ using *M*_0_) to predict future event risk (note that the model is also static in this setting, though we will call it *static scores* for brevity). We observed that the static scores performed reasonably well even 2-3 years after *t*_0_, although discrimination and calibration were gradually lost (Supplementary Fig. 4a–c). More generally, we observe that scores fitted and calculated at a fixed time cutoff had successively lower AUROCs for predicting EA over future periods (Supplementary Fig. 4d). Although the absolute differences in AUROC over time with static scores are small, they are visibly larger than those seen between SPARRA*v*3 and SPARRA*v*4 (Fig. 2a), indicating that comparisons analogous to Fig. 2e, f would similarly show much larger differences. This affirms the need for updated scores in deployment, despite the static model.

### Feature importance

**Fig. 5:**
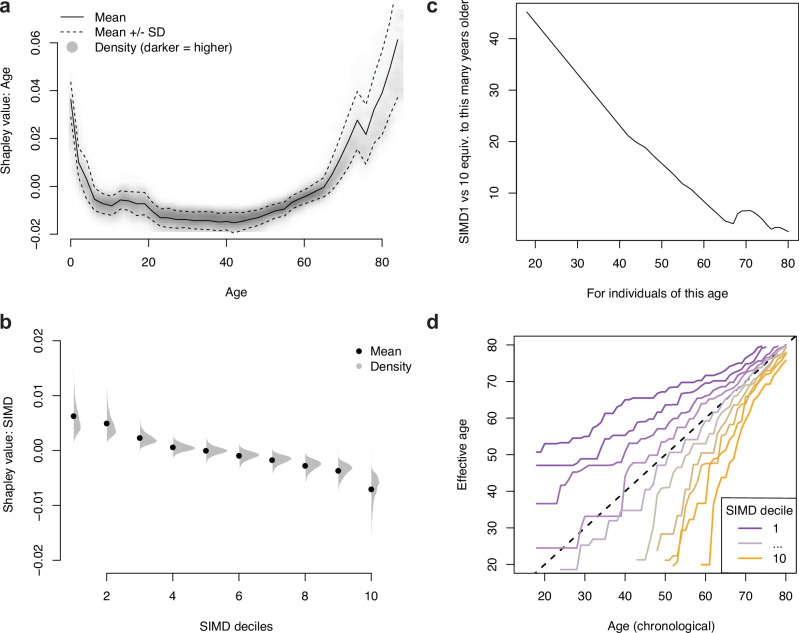
Analysis of Shapley values. Distribution of Shapley values by (**a**) age and (**b**) SIMD deciles (1: most deprived; 10: least deprived). **c** Number of additional years of age needed to match the difference in Shapley values between SIMD deciles 1 and 10. **d** `Effective ages' calculated to match EA rates: for an (age, SIMD decile) pair, the age at mean SIMD with the equivalent EA rate.

The features with the largest mean absolute Shapley value (excluding SPARRA*v*3 and the features derived from the topic model) were age, the number of days since the last EA, the number of previous A&E attendances, and the number of antibacterial prescriptions (Table 3). Most features had non-linear effects (see e.g. Supplementary Fig. 5a-b). For example, the risk contribution from age was high in infancy, dropping rapidly from infancy through childhood, then remaining stable until around age 65, and rising rapidly thereafter (Fig. 5a). We also found a non-linear importance of SIMD (Fig. 5b) and number of previous emergency hospital admissions (Supplementary Fig. 5c).

**Table 3:** Top 20 most important variables by mean absolute Shapley value (percentage scale)

Variable	Importance
Age at time cutoff	1.530
Days since last emergency admission	0.752
Number of previous A&E attendances	0.509
Number of antibacterial prescriptions	0.376
Number of central nervous system related prescriptions	0.375
Male sex	0.373
Days since last A&E attendance	0.321
SIMD decile	0.310
Number of emergency bed days	0.299
Days since last acute admission of any type	0.285
Days since last outpatient attendance	0.257
Number of diuretic prescriptions	0.213
Number of lipid lowering drug prescriptions	0.194
Number of previous first outpatient appointments	0.190
Number of recorded long term conditions	0.173
Number of emergency admissions	0.161
Total number of filled prescriptions	0.160
Number of antianaemic prescriptions	0.159
Number of bronchodilator prescriptions	0.152
Number of BNF sections from which a prescription was filled	0.141

Importance can be interpreted as the average percent added or subtracted to risk score due to this factor.

We further investigated the contribution of SIMD by comparing Shapley values between features. We computed the mean difference in contribution of SIMD to risk score between individuals in the most deprived and least deprived SIMD decile areas, and the additional years of age which would contribute an equivalent amount. This was generally around 10-40 additional years (Fig. 5d). In terms of raw admission rates, disparity was further apparent: individuals aged 20 in lowest SIMD decile areas had similar admission rates to individuals aged 70 in the 3 highest SIMD decile areas (Fig. 5e).

When exploring the added value (in terms of AUROC) of including the features derived using the topic model (Supplementary Table 4), we observed slightly better performance than the model without such features (*p*-value = 3 × 10^−29^; Supplementary Fig. 5e, f). In some cases, topic features led to substantial changes in overall score: for example, a topic relating to skin disease contributed more than 2% to the SPARRA*v*4 score (roughly equivalent to the mean contribution to the score from age for individuals aged 75; see Fig. 5a) for around 0.43% of individuals with the resultant SPARRA*v*4 scores better-calibrated than the SPARRA*v*3 scores, which did not use a topic model (Supplementary Note 1). Analogously to Fig. 2e, we also computed the additional number of samples correctly identified as having an event amongst the top scores by the two models. Although the absolute difference in AUROC was small, we found that the use of topic features increased the number of EAs detected in the top 500,000 scores by around 200.

### Deployment

SPARRA*v*4 was developed in a remote data safe haven (DSH) environment^27^ without access to internet or modern collaboration tools (e.g. git version control). Whilst our analysis code and a summary of model outputs (e.g. AUROC values) could be securely extracted from the DSH, this was not possible for the actual trained model due to potential leaks of sensitive patient information^28^. This introduced reproducibility challenges, since the model had to be retrained in a different secure environment before it was deployed by PHS. In particular, this re-development outside the DSH had two distinct phases. Firstly, the raw data transformations (to convert the original databases into a format that is suitable for ML algorithms) were reproduced from scratch from the same source data. Once the output of the transformations matched perfectly between the DSH and the external environment for all features, the topic and predictive models were re-trained. The training process could not be exactly matched due to differing compute environments, package versions and training/validation split. However, after training, the external models were validated by comparing the performance (via AUROC) and the calibration with the results obtained within the DSH.

**Fig. 6:**
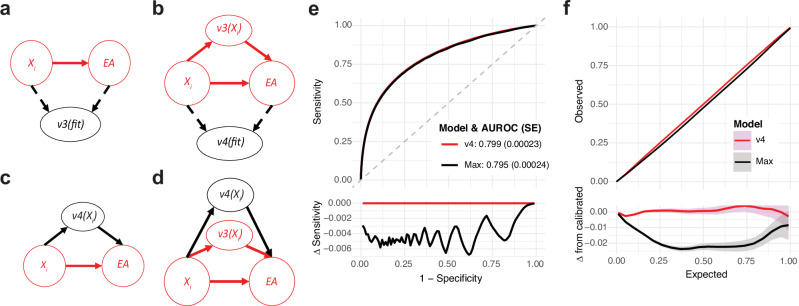
Model updating in the presence of performative effects. **a**, **d** Causal structure for the training and deployment of SPARRA*v*3 and SPARRA*v*4. *X*_*i*_ represents covariates for a patient-time pair; *v*3(*fit*)/*v*4(*fit*) and *v*3(*X*_*i*_)/*v*4(*X*_*i*_) represent the fitting and deployment of *v*3 and *v*4 respectively. **a** Training setting for SPARRA*v*3. **b** Training setting for SPARRA*v*4. **c** Deployment setting if SPARRA*v*4 were to naively replace SPARRA*v*3. **d** Deployment setting in which SPARRA*v*4 is used as an adjuvant to SPARRA*v*3. **e** Comparison of discrimination (ROC) between SPARRA*v*4 and the maximum of both scores. **f** Comparison of calibration between SPARRA*v*4 and the maximum of both scores.

Another practical issue that arises when developing and deploying a new version of SPARRA is due to potential *performative prediction* effects^29^. Since SPARRA*v*3 is already visible to GPs (who may intervene to reduce the risk of high-risk patients), *v*3 can alter observed risk in training data used for *v*4, with *v*3 becoming a *‘victim of its own success’*^30,31^. This is potentially hazardous: if some risk factor *R* confers high *v*3 scores prompting GP intervention (e.g., enhanced follow-up), then in the training data for *v*4, *R* may no longer apparently confer increased risk. Should *v*4 replace *v*3, some individuals would therefore have their EA risk underestimated, potentially diverting important anticipatory care away from them. This highlights a critical problem in the theory of model updating^32^, which we expand on in Methods and illustrate in Fig. 6a–d. As a practical solution, during deployment, GPs could receive the maximum between *v*3 and *v*4 scores. This would avoid the potential hazard of risk underestimation, at the cost of mild loss of AUROC (Fig. 6e) and score calibration (Fig. 6f).

## Discussion

We used routinely collected EHRs from around 5.8 million Scottish residents to develop and evaluate SPARRA*v*4, a risk score that quantifies 1-year EA risk based on age, deprivation (using SIMD as a geographic-based proxy) and a wide range of features derived from a patient’s past medical history. SPARRA*v*4 constitutes a real-world use of ML, derived from population-level data and embedded in clinical settings across Scotland (Fig. 1).

While the increases in AUROC and AUPRC over the previous version of SPARRA may be small (Fig. 2a, b), the improvement provided by SPARRA*v*4 in terms of absolute benefit to population is substantial (Fig. 2e, f). This arises from the use of more flexible ML methods (e.g. to capture non-linear patterns between features and EA risk) and the incorporation of features derived by a topic model which extracts more granular information (with respect to the manually curated features used by SPARRA*v*3) from past diagnoses and prescriptions data. The latter can be thought of as a proxy for multi-morbidity patterns, in that topic models identify patterns of diagnoses and prescriptions which commonly occur together^33^, which can be seen to occur in our data (Supplementary Table 4). The use of an ensemble of models also allows stronger models and methods to dominate the final predictor, and weaker models to be discarded.

Our analysis also provides insights into the epidemiology of EA risk, highlighting predictable patterns in terms of EA type (as defined by the recorded primary diagnosis; Fig. 3b) and the imminence of EA (Fig. 3c), in that those at high risk of an admission are likely to have an imminent admission rather than equally likely to have an admission over the year-long prediction period. Moreover, we studied the contribution of each feature, revealing a complex relationship between age, deprivation and EA risk (Fig. 5). Note, however, that we cannot assign a causal interpretation for any reported associations. In particular, the link between SIMD and EA risk is complex; SIMD includes a ‘health’ constituent^24^, and individuals in more-deprived SIMD decile areas (1: most deprived; 10: least deprived) miss more primary care appointments^34^.

One important strength of SPARRA*v*4 is its nationwide coverage, using existing healthcare databases without the need for additional bespoke data collection. This, however, prevents the use of primary care data (beyond community prescribing) as it is not currently centrally collected in Scotland. Due to privacy considerations, we were also unable to access geographic location data, precluding the study of potential differences between e.g. rural and urban areas and the use of a geographically separated test set^8^. Limited data availability also limits a straightforward comparison of predictive performance (e.g. in terms of AUROC) with respect to similar models developed in England^6,10^ (this is also complicated because of different model choices, e.g.^6^ modelled time-to-event data but we used a binary 1-year EA indicator). For example, we do not have information about marital and smoking status, blood test results and family histories; all of which were found to be predictive of EA risk by Ref. ^6^. Our training dataset is non-representative of our raw dataset (which in turn is non-representative of the Scottish population, as per Table 1, as is typical of studies based on electronic health records^35,36^), but it does generally include individuals at higher EA risk.

Beyond model development and evaluation, our work also highlights broader challenges that arise in this type of translational project using EHR. In particular, as SPARRA*v*4 has the potential to influence patient care, we have placed high emphasis on transparency and reproducibility while ensuring compliance with data governance constraints. Providing our code in a publicly available repository will also allow us to transparently document future changes to the model (e.g. if any unwanted behaviour is identified during the early stages of deployment). SPARRA*v*4 also constitutes a real-world example in which potential performative effects need to be taken into account when updating an already deployed risk prediction model (Fig. 6).

It is critical to emphasise that SPARRA*v*4 will not replace clinical judgement, nor does it direct changes to patient management made solely based on the score. Indeed, any potential interventions must be decided jointly by medical professionals and patients, balancing the underlying risks and benefits. Moreover, lowering EA risk does not necessarily entail overall patient benefit as e.g. long-term oral corticosteroid use in mild asthmatics would reduce EA risk, but the corticosteroids themselves can cause an unacceptable cost of long-term morbidity^37^.

Optimal translation into clinical action is a vital research area and is essential for quantifying the benefit of such scores in clinical practice. Indeed, any benefit is dependent on widespread uptake and the existence of timely integrated health and social care interventions, and identification of EA risk is only the first step in this pathway. As such, the evaluation of real-world effectiveness for SPARRA*v*4 and similar risk scores is complex, and requires a multi-disciplinary approach that considers a variety of factors (e.g. the local health economy and the capacity to deliver pre-emtive interventions in primary care). Therefore, we will continue to collaborate to achieve successful deployment of SPARRA*v*4 and will carefully consider the feedback from GPs to improve the model and the communication of its results further (e.g. via informative dashboards). As the COVID-19 pandemic resolves, it will also be important to assess potential effects of dataset shift^38^ due to disproportionate mortality burden in older individuals and long-term consequences of COVID-19 infections. In an era where healthcare systems are under high stress, we hope that the availability of robust and reproducible risk scores such as SPARRA*v*4 (and its future developments) will contribute to the design of proactive interventions that reduce pressures on healthcare systems and improve healthy life expectancy.

## Methods

### Ethics and data governance

The project was covered under National Safe Haven Generic Ethical Approval (favourable ethical opinion from the East of Scotland NHS Research Ethics Service). This study was conducted in accordance with UK data governance regulations and the use of patient-level EHR was approved by the Public Benefit and Privacy Panel (PBPP) for Health and Social Care (study number 1718-0370; approval evidenced in application outcome minutes for 2018/19 at https://www.informationgovernance.scot.nhs.uk/pbpphsc/application-outcomes/). Data access was also approved by the PHS National Safe Haven, through the electronic Data Research and Innovation Service (eDRIS).

All studies have been conducted in accordance with information governance standards; data had no patient identifiers available to the researchers. Due to the confidential nature of the data, all analysis took place on a remote “data safe haven”, without access to internet, software updates or unpublished software. Information Governance training was required for all researchers accessing the analysis environment. Moreover, to avoid the risk of accidental disclosure of sensitive information, an independent team carried out statistical disclosure control checks on all data exports, including the outputs presented in this manuscript.

### SPARRA*v*3

SPARRA*v*3^12^, deployed in 2012, uses separate logistic regressions on four subcohorts of individuals: frail elderly conditions (FEC; individuals aged > 75); long-term conditions (LTC; individuals aged 16–75 with prior healthcare system contact), young emergency department (YED; individuals aged 16-55 who have had at least one A&E attendance in the previous year) and under-16 (U16; individuals aged < 16). If an individual belongs to more than one of these groups, the maximum of the associated scores is reported. SPARRA*v*3 was fitted once (at its inception in 2012) with regression coefficients remaining fixed thereafter. Most input features were manually dichotomised into two or more ranges for fitting and prediction. The prediction target for SPARRA*v*3 is EA within 12 months. People who died in the pre-prediction period, and who therefore do not have an outcome for use in the analysis, are excluded. PHS calculated SPARRA*v*3 scores and provided them as input for the analysis described herein. Any GP in Scotland can access SPARRA scores after attaining information governance approval.

### Exclusion criteria

The exclusion criteria were applied per sample (defined as individual-time pairs; Fig. 1c). Samples were excluded if: (i) they were excluded from SPARRA*v*3 (these are individuals for which PHS did not calculate a SPARRA*v*3 score and largely correspond to individuals with no healthcare interactions or that were not covered by the four SPARRA*v*3 subcohorts^12^;), (ii) when the individual died prior to the prediction time cutoff, (iii) when the SIMD for the individual was unknown, or (iv) those associated to individuals whose Community Health Index (CHI ^39^) changed during the study period (‘Unmatched’ in Fig. 1). The CHI number is a unique identifier which is used in Scotland for health care purposes. Rates of EA and death in the follow-up period were generally lower in excluded samples than in included samples (3.40% versus 8.88%, only considering exclusions which were not due to the individual having died prior to the time cutoff; Supplementary Table 6). Exclusion criteria (i) and (ii) were applied at the sample level, while exclusion criteria (iii) and (iv) were applied at the individual level.

### Feature engineering

A typical entry in the source EHR tables (Supplementary Table 2) recorded a single interaction between a patient and NHS Scotland (e.g. hospitalisation), comprising a unique individual identifier (an anonymised version of the CHI number), the date on which the interaction began (admission), the date it ended (discharge), and further details (diagnoses made, procedures performed). For each sample, entries from up to three years before the time cutoff were considered when building input features, except long-term condition (LTC) records, which considered all data since recording began in January 1981. A full feature list is described in Supplementary Table 3. This includes SPARRA*v*3^12^ features, e.g. age, sex, SIMD deciles and counts of previous admissions (e.g. A&E admissions, drug-and-alcohol-related admissions). Additional features encoding time-since-last-event (e.g. days since last outpatient attendance) were included following findings in Ref. ^6^. From community prescribing data, we derived predictors encoding the number of prescriptions of various categories (e.g. respiratory), extending the set of predictors beyond a similar set used in SPARRA*v*3. Similarly to SPARRA*v*3, we also derived the total number of different prescription categories, the total number of filled prescription items, and the number of British National Formulary (BNF) sections from which a prescription was filled^40^. From LTC records, we extracted the number of years since diagnosis of each LTC (e.g. asthma), the total number of LTCs recorded, and the number of LTCs resulting in hospital admissions.

Data from prescription records and recorded diagnoses tend to be sparse, in that most medications and diagnoses will only be recorded for a small proportion of the population. We used our topic model^20^ to assimilate this data, by jointly modelling prescriptions and diagnoses using 30 topics (effectively clusters of prescriptions and diagnoses), considering samples as ‘documents’ and diagnoses/prescriptions as ‘words’. This enabled a substantial reduction in feature dimensionality, given the number of diagnoses/prescription factor levels. Using the map from documents to topic probabilities, we used derived topic probabilities as additional features in SPARRA*v*4, which corresponded to sample-wise membership of each topic.

### Choice of prediction target for SPARRA*v*4

The primary target for SPARRA is to predict whether an individual will experience an EA within 12 months from the prediction cutoff. A problem arises due to the deaths during the follow-up year for which the target may be unknown (e.g. if someone died within 6 months, without a prior EA). Broadly, there are four options for how to treat such individuals during model training and testing:

1. Exclude them from the dataset
2. Treat them according to whether they had an EA before they died
3. Treat them as no EA
4. Treat them as an EA

It would also be possible to code death in follow-up differentially; for instance, coding in-hospital death as EA and in-community death as exclusions or non-EA. Our choice not to code all deaths identically is in the interests of non-maleficence. If an individual is at risk of imminent death in the community they will typically be admitted to hospital if it is possible to react in time, with a possible exemption if this is not in their best interests.

Option 1 would exclude the most critically ill individuals from the dataset and hence was discarded. Option 2 would effectively mean such individuals have a follow-up time less than a year, and would classify individuals who died without a hospital admission as having had a ‘desirable’ outcome. Option 3 would effectively classify death as a ‘desirable’ outcome, so we avoided it. The consequences from coding community deaths as non-EA would be severe, as it could mean that healthier individuals at risk of sudden death are either coded as non-EA or excluded from the dataset, potentially leading to inappropriately low scores being assigned to these individuals. This could draw treatment away from individuals in high need. Instead, option 4 allows the general description of the target as ‘a catastrophic breakdown in health’. In this case, our model would not be able to distinguish community deaths from emergency admissions: we may assign high ’EA’ scores to the very old and terminally ill, when in fact these individuals may be treated in the community rather than admitted. The potential harm from this option is small. It could mean that such individuals are excessively treated rather than palliated, but since palliation over treatment is an active decision^41^ and such individuals are generally known to be high-risk it is unlikely that the SPARRA score will adversely affect any decisions in this case. As the philosophy of the SPARRA score is to avert breakdowns in health, of which death can be considered an example, we decided to use a composite prediction target (EA or death within 12 months) which is consistent with option 4.

### ML prediction methods

For SPARRA*v*4, we had no prior belief that any ML model class would be best, so considered a range of binary prediction approaches (hereafter referred to as constituent models). The following models were fitted using the h2o^42^ R package (version 3.24.0.2): an artificial neural network (ANN), two random forests (RF) (depth 20 and 40), an elastic net generalised linear model (GLM) and a naive Bayes (NB) classifier. The xgboost^43^ R package (version 1.6.0.1) was used to train three gradient-boosted trees (XGB) models (maximum tree depth 3, 4, and 8). Hyper-parameter choices are described in Methods. SPARRA*v*3 was used as an extra constituent model.

Rather than selecting a single constituent model, we used an ensemble approach. Similar to^19^, we calculated an optimal linear combination (*L*_1_-penalised regression, using the R package glmnet, version 4.1.4) of the scores generated by each constituent model. Ensemble weights were chosen to optimise the AUROC. Finally, we monotonically transformed the derived predictor to improve calibration by inverting the empirical calibration function (Supplementary Note 2).

### Data imputation

As all non-primary care interactions with NHS Scotland are recorded in the input databases, there was no missingness for most features. For ‘time-since-interaction’ type features, samples for which there was no recorded interaction were coded as twice the maximum lookback time. There was minor non-random missingness in topic features (~ 0.8%) due to individuals in the dataset with no diagnoses or filled prescriptions, for whom topic probabilities could not be calculated. We used mean-value imputation in the ANN and GLM models (deriving mean values from training data only), used missingness to inform tree splits (defaults in Ref. ^42^) in RF, used sample-wise imputation in XGB (as per^43^) and dropped during fitting (default in Ref. ^42^) in NB (omitted missing values for prediction). All imputation rules were determined using training sets only.

Particular care was required for features encoding total lengths of hospital stays. In some cases, a discharge date was not recorded, which could lead to an erroneous assumption of a very long hospital stay (from admission until the time cutoff). To address this, we truncated apparently spuriously long stays at data-informed values (Supplementary Note 4).

### Hyperparameter choice for ML prediction methods

We used a range of constituent models. Unless otherwise specified, hyperparameters were set as the software defaults. When tuned, hyperparameter values were chosen to optimise the default objective functions implemented for each method: log-loss or the ANN, RFs and GLM, likelihood for the NB model; and a logistic objective for the XGB trees. In all cases, hyperparameters were determined by randomly splitting the relevant dataset into a training and test set of 80% and 20% of the data respectively. Details for each method are provided below. Only limited hyperparameter tuning was possible due to the restricted computational environment in the data safe haven (see Results).

#### SPARRA*v*3

SPARRA*v*3 scores were calculated by PHS using their existing algorithm^12^.

#### Artificial neural network (ANN)

We used a training dropout rate of 20% to reduce generalisation error. We optimised over the number of layers (1 or 2) and the number of nodes in each layer (128 or 256).

#### Random forest (RF)

We fitted two RF: one had maximum depth 20 and 500 trees, and the other had maximum depth 40 and 50 trees (both taking a similar time to fit).

#### Gradient-boosted trees (XGB)

We fitted three boosted tree models with three maximum depths: 3, 4, and 8. For the deeper-tree model, we set a low step size shrinkage *η* = 0.075 and a positive minimum loss reduction *γ* = 5 in order to avoid overfitting. In the other two models, we used default values of *η* = 0.3, *γ* = 0.

#### Naive Bayes (NB)

The only hyperparameter we tuned was a Laplace smoothing parameter, varying between 0 and 4.

#### Penalised Generalised linear model (GLM)

We optimised *L*_1_ and *L*_2_ penalties (an elastic net), considering total penalty (*L*_1_ + *L*_2_) in 10^−{1, 2, 3, 4, 5}^, and a ratio *L*_1_/*L*_2_ in {0, 0.5, 1}.

### Cross-validation

We fitted and evaluated SPARRA*v*4 using three-fold cross-validation (CV). We considered three-fold cross validation acceptable in our case given the size of our dataset^44^. This was designed such that all elements of the model evaluated on a test set were agnostic to samples in that test set. Individuals were randomly partitioned into three data folds (F1, F2 and F3). At each CV iteration, F1 and F2 were combined and used as a training dataset, F3 was used as a test dataset. The training dataset (F1+F2) was used to fit the topic model and to train all constituent models (except SPARRA*v*3, whose training anyhow pre-dates the data used here). The ensemble weights and re-calibration transformation were learned using F1 + F2, i.e. without using the test set from the test set (Supplementary Note 2).

### Predictive performance

Our primary endpoint for model performance was AUROC. We also considered area-under-precision-recall curves (PRC) and calibration curves. We plotted calibration curves using a kernelised calibration estimator (Supplementary Note 5).

For simplicity, figures show ROC/PRC that were calculated by combining all samples from the three *test* CV folds (that is, all scores and observed outcomes were merged to draw a single curve). Quoted AUROC/AUPRC values were calculated as an average across the three *test* CV folds to avert problems from between-fold differences in models^45^. For ease of comparison, we also used mean-over-folds to compute quoted AUROCs and AUPRCs for SPARRA*v*3, although the latter was not fitted to our data.

### Deployment scenario stability and performance attenuation

Using the same analysis pipeline as for the development of SPARRA*v*4, we trained a static model *M*_0_ to an early time cutoff (*t*_0_=1 May 2014), and using one year of data prior to *t*_0_ to derive predictors (the restricted lookback is the only deviation from the actual model pipeline, due to limited temporal span of the training data).

We studied the performance of *M*_0_ as a *static model* to repeatedly predict risk at future time cutoffs, which mirrors the way in which PHS will deploy the model. To do this, we assembled test features from data 1 year prior to *t*_1_=1 May 2015, *t*_2_=1 Dec 2015, *t*_3_=1 May 2016, *t*_4_=1 Dec 2016, and *t*_5_=1 May 2017, applying *M*_0_ to predict EA risk in the year following each time-point. In this analysis, the comparison of the distribution of scores over time only considered the cohort of patients who were alive and had valid scores at *t*_1_, …, *t*_5_.

To ensure a fair comparison when evaluating the performance of *static scores* (computed at *t*_0_ using *M*_0_) to predict future event risk (at *t*_1_, …, *t*_5_), we only considered a subsample of 1 million individuals with full data across all time-points, selected such that global admission rates matched those at *t*_0_.

### Assessment of feature importance

We examined the contribution of feature to risk scores at an individual level by estimating Shapley values^21^ for each feature. For simplicity, this calculation was done using 20,000 randomly-chosen samples in the first cross-validation fold (F1). We treated SPARRA*v*3 scores as fixed predictors rather than as functions of other predictors.

We also assessed the added value of inclusion of topic-model derived features, which summarise more granular information about the previous medical history of a patient with respect to those included in SPARRA*v*3. For this purpose, we refitted the model to F2+F3 with topic-derived features excluded from the predictor matrix. We compared the performance of these models using F1 as test data. We compared the performance of predictive models with and without the features derived from the topic model by comparing AUROC values using DeLong’s test^46^.

### Model updating in the presence of performative effects

We aim to produce the SPARRA score to accurately estimate EA risk over a year under normal medical care. In other words, the score should represent the EA risk if GPs do not already have access to such a risk score. Because GPs see a SPARRA score (SPARRA*v*3) and may act on it, the observed risk may be lower than predicted - the score may become a ‘victim of its own success’^30,31^ due to performative effects^29^. Unfortunately, since the SPARRA*v*3 score is widely available to Scottish GPs, and may be freely acted on, we cannot assess the behaviour of the medical system in its absence. This is potentially hazardous^32^.

Formally, at a given fixed time, for each individual, the value of ‘EA in the next 12 months’ is a Bernoulli random variable. The probability of the event for individual *i* is conditional on a set of covariates *X*_*i*_ derived from their EHR. We denote *v*3(*X*_*i*_), *v*4(*X*_*i*_) the derived SPARRA*v*3 and SPARRA*v*4 scores as functions of covariates, and assume a causal structure shown in Fig. 6 (for simplicity, we assume there are no unobserved confounders but the same argument applies in their presence). With no SPARRA-like predictive score in place, there is only one causal pathway *X*_*i*_ → *EA*. It is to this system (coloured red) that *v*3 was fitted. Here, *v*3(*X*_*i*_) estimates the ‘native’ risk *P*(*EA* | *X*_*i*_) (ignoring previous versions of the SPARRA score, which covered < 30% of the population). Although *v*3(*X*_*i*_) is determined entirely by *X*_*i*_, the act of distributing values of *v*3(*X*_*i*_) to GPs opens a second causal pathway from *X*_*i*_ to *EA* (Fig. 6) driven by GP interventions made in response to *v*3(*X*_*i*_) scores. It is to this system (coloured red) that SPARRA*v*4 is fitted. Hence, *v*4(*X*_*i*_) is an estimator of *P*(*EA* | *X*_*i*_, *v*3(*X*_*i*_)), a ‘conditional’ risk after interventions driven by *v*3(*X*_*i*_) have been implemented.

If SPARRA*v*4 naively replaced SPARRA*v*3 (Fig. 6), we would be using *v*4(*X*_*i*_) to predict behaviour of a system different to that on which it was trained (Fig. 6). To amend this problem, we propose to use SPARRA*v*4 in *conjunction* with SPARRA*v*3 rather than to completely replace it (Fig. 6). Ideally, GPs would be given *v*3(*X*_*i*_) and *v*4(*X*_*i*_) simultaneously and asked to *firstly* observe and act on *v*3(*X*_*i*_), *then* observe and act on *v*4(*X*_*i*_), thereby only using *v*4(*X*_*i*_) as per Fig. 6. This is impractical, so instead, we propose to distribute a single value (given by the maximum between *v*3(*X*_*i*_) and *v*4(*X*_*i*_)), avoiding the potential hazard of risk underestimation, at the cost of mild loss of score calibration (Fig. 6).

## Supplementary information

Supplemental material

## Data Availability

Raw data for this project are patient-level EHR, which have been anonymised for confidentiality ahead of any analysis being undertaken. Enquiries about access to this data may be directed to phs.edris@phs.scot. However, the summary data required to draw figures included in our manuscript is publically available from our GitHub repository. All publicly available data summaries were reviewed by an independent team to avoid the risk of accidental disclosure of sensitive information.

## Code availability

All analysis code and co-ordinates required to reproduce our Figures are available in github.com/jamesliley/SPARRAv4 This manuscript conforms to the TRIPOD guidelines^23^ (Supplementary Table 1).
