# Publisher Correction: Development and assessment of a machine learning tool for predicting emergency admission in Scotland

**DOI:** 10.1038/s41746-024-01310-6

**Published:** 2024-10-26

**Authors:** James Liley, Gergo Bohner, Samuel R. Emerson, Bilal A. Mateen, Katie Borland, David Carr, Scott Heald, Samuel D. Oduro, Jill Ireland, Keith Moffat, Rachel Porteous, Stephen Riddell, Simon Rogers, Ioanna Thoma, Nathan Cunningham, Chris Holmes, Katrina Payne, Sebastian J. Vollmer, Catalina A. Vallejos, Louis J. M. Aslett

**Affiliations:** 1 Department of Mathematical Sciences, Durham University, Durham, UK; 2 Alan Turing Institute, London, UK; 3 MRC Human Genetics Unit, Institute of Genetics and Cancer, University of Edinburgh, Edinburgh, UK; 4 Mathematics Institute, University of Warwick, Coventry, UK; 5 Institute of Health Informatics, University College London, London, UK; 6 Wellcome Trust, London, UK; 7 Public Health Scotland (PHS), Edinburgh, UK; 8 University of St Andrews, St Andrews, UK; 9 NHS National Services Scotland, Edinburgh, UK; 10 Department of Statistics, University of Warwick, Coventry, UK; 11 Department of Statistics, University of Oxford, Oxford, UK; 12 University of Kaiserslautern-Landau, Kaiserslautern, Germany; 13 German Research Centre for Artificial Intelligence, Kaiserslautern, Germany

**Keywords:** Public health, Statistics

**Correction to:** *npj Digital Medicine* 10.1038/s41746-024-01250-1, published online 23 October 2024

In this article the affiliation details for Author Louis J. M. Aslett were incorrectly given as ‘^2^Alan Turing Institute, London, UK. ^3^MRCHuman Genetics Unit, Institute of Genetics and Cancer, University of Edinburgh, Edinburgh, UK.’ but should have been ‘^1^Department of Mathematical Sciences, Durham University, Durham, UK. ^2^Alan Turing Institute, London, UK’. The original article has been corrected.

