# Supplementary material for: Development and assessment of a machine learning tool for predicting emergency admission in Scotland
Source: NPJ Digit Med. 2024 Oct 23;7:277. doi: 10.1038/s41746-024-01250-1 (PMC11499905; doi:10.1038/s41746-024-01250-1)
Supplement: Supplementary file 1 — Supplemental material [file 41746_2024_1250_MOESM1_ESM.pdf]

Development and assessment of a machine learning tool for predicting emergency admission in Scotland

James Liley[1,2,3,⊥,*], Gergo Bohner[1,4,⊥], Samuel R. Emerson[3], Bilal A. Mateen[1,5], Katie Borland[6], David Carr[6], Scott Heald[6], Samuel D. Oduro[6b], Jill Ireland[6], Keith Moffat[6,7], Rachel Porteous[6], Stephen Riddell[6b], Simon Rogers[8], Ioanna Thoma[1,2], Nathan Cunningham[1,9], Chris Holmes[1,10], Katrina Payne[1], Sebastian J. Vollmer[1,4], Catalina A. Vallejos[1,2,*,†], and Louis J. M. Aslett[1,3,*,†]

[1]Alan Turing Institute, London, UK
[2]MRC Human Genetics Unit, Institute of Genetics and Cancer, University of Edinburgh, UK
[3]Department of Mathematical Sciences, Durham University, UK
[4]Mathematics Institute, University of Warwick, UK
[5]Institute of Health Informatics, University College London, UK, and Wellcome Trust, London, UK
[6]Public Health Scotland (PHS). (b): former employee
[7]University of St Andrews, UK
[8]NHS National Services Scotland, UK
[9]Department of Statistics, University of Warwick, UK
[10]Department of Statistics, University of Oxford, UK
[⊥]Equal contribution
[†]Equal supervision
[*]Corresponding

ABSTRACT

Emergency admissions (EA), where a patient requires urgent in-hospital care, are a major challenge for healthcare systems. The development of risk prediction models can partly alleviate this problem by supporting primary care interventions and public health planning. Here, we introduce SPARRA*v*4, a predictive score for EA risk that will be deployed nationwide in Scotland. SPARRA*v*4 was derived using supervised and unsupervised machine-learning methods applied to routinely collected electronic health records from approximately 4.8M Scottish residents (2013-18). We demonstrate improvements in discrimination and calibration with respect to previous scores deployed in Scotland, as well as stability over a 3-year timeframe. Our analysis also provides insights about the epidemiology of EA risk in Scotland, by studying predictive performance across different population sub-groups and reasons for admission, as well as by quantifying the effect of individual input features. Finally, we discuss broader challenges including reproducibility and how to safely update risk prediction models that are already deployed at population level.

Keywords: Emergency admission, Primary care, Machine learning

CONTENTS

Summary **S2**

Supplementary Tables **S3**

Supplementary Figures **S18**

Supplementary Notes **S24**

1 Analysis of specific topic effects . . . S24
2 Model re-calibration . . . S24
3 Use of subcohort-specific ensemble coefficients . . . S25
4 Imputation of lengths of stay when discharge date was missing . . . S25
5 Assessment of calibration . . . S26

SUMMARY

Supplementary Table 1 Checklist for TRIPOD guidelines [1].

Supplementary Table 2 Definition of input features for SPARRAv4

Supplementary Table 3 Definition of input features for SPARRA*v*4.

Supplementary Table 4 Exploration of contributors to each topic.

Supplementary Table 5 Definition of different admission types.

Supplementary Table 6 Frequency of admissions and deaths amongst excluded samples.

Supplementary Table 7 Coefficients of ensemble when fitted separately to SPARRA*v*3 cohorts.

Supplementary Figure 1 Extended data overview.

Supplementary Figure 2 Density plot comparing SPARRA*v*3 and SPARRA*v*4 scores.

Supplementary Figure 3 Calibration curves for SPARRA*v*4 model constituents.

Supplementary Figure 4 Performance of a static model and static scores used to predict risk at future time cutoffs.

Supplementary Figure 5 Feature importance

Supplementary Note 1 Analysis of specific effects of a topic feature.

Supplementary Note 2 Details of the re-calibration procedure.

Supplementary Note 3 Investigation of use of SPARRA*v*3-cohort specific ensemble coefficients

Supplementary Note 4 Imputation of lengths of stay when discharge date was missing.

Supplementary Note 5 Assessment of calibration.

SUPPLEMENTARY TABLES

Section	Item	Checklist item	Section	Page
Title and abstract				
Title	1	Identify the study as developing and/or validating a multivariable prediction model, the target population, and the outcome to be predicted.	Title	-
Abstract	2	Provide a summary of objectives, study design, setting, participants, sample size, predictors, outcome, statistical analysis, results, and conclusions.	Abstract	-
Introduction				
Background and objectives	3a	Explain the medical context (including whether diagnostic or prognostic) and rationale for developing or validating the multivariable prediction model, including references to existing models.	Introduction	3
	3b	Specify the objectives, including whether the study describes the development or validation of the model or both.	Introduction	3
Methods				
Source of data	4a	Describe the study design or source of data (e.g., randomized trial, cohort, or registry data), separately for the development and validation data sets, if applicable.	M: Overview and cohort details; M: Feature engineering	4, 10
	4b	Specify the key study dates, including start of accrual; end of accrual; and, if applicable, end of follow-up.	M: Overview and cohort details	4
Participants	5a	Specify key elements of the study setting (e.g., primary care, secondary care, general population) including number and location of centres.	M: Feature engineering	10
	5b	Describe eligibility criteria for participants.	M: Overview and cohort details	4
	5c	Give details of treatments received, if relevant.	-	-
Outcome	6a	Clearly define the outcome that is predicted by the prediction model, including how and when assessed.	M: Overview and cohort details	4
	6b	Report any actions to blind assessment of the outcome to be predicted.	-	-
Predictors	7a	Clearly define all predictors used in developing or validating the multivariable prediction model, including how and when they were measured.	M: Feature engineering, table of predictors	10, Supplementary Table 3
	7b	Report any actions to blind assessment of predictors for the outcome and other predictors.	-	-
Sample size	8	Explain how the study size was arrived at.	Introduction	4
Missing data	9	Describe how missing data were handled (e.g., complete-case analysis, single imputation, multiple imputation) with details of any imputation method.	Supp: Missing values	10

Statistical analysis methods	10a	Describe how predictors were handled in the analyses.	M: Machine learning prediction methods	13
	10b	Specify type of model, all model-building procedures (including any predictor selection), and method for internal validation.	M: Machine learning prediction methods; M: Cross-validation	13,14
	10c	For validation, describe how the predictions were calculated.	M: Cross-validation	14
	10d	Specify all measures used to assess model performance and, if relevant, to compare multiple models.	M: Predictive performance	15
	10e	Describe any model updating (e.g., recalibration) arising from the validation, if done.	M: Model updating	18
Risk groups	11	Provide details on how risk groups were created, if done.	-	-
Development vs. validation	12	For validation, identify any differences from the development data in setting, eligibility criteria, outcome, and predictors.	D: Relation to other studies	9
**Results**				
Participants	13a	Describe the flow of participants through the study, including the number of participants with and without the outcome and, if applicable, a summary of the follow-up time. A diagram may be helpful.	M: Feature engineering, figure 1	10, figure 1
	13b	Describe the characteristics of the participants (basic demographics, clinical features, available predictors), including the number of participants with missing data for predictors and outcome.	M: Overview and cohort details; M: Feature engineering, figure 1	4, 10, figure 1
	13c	For validation, show a comparison with the development data of the distribution of important variables (demographics, predictors and outcome).	Not applicable; see discussion	Not applicable; see page 9
Model development	14a	Specify the number of participants and outcome events in each analysis.	M: Overview and cohort details	4
	14b	If done, report the unadjusted association between each candidate predictor and outcome.	-	-
Model specification	15a	Present the full prediction model to allow predictions for individuals (i.e., all regression coefficients, and model intercept or baseline survival at a given time cutoff).	Not possible: see code and data availability note	Not possible: see page 19
	15b	Explain how to the use the prediction model.	D: Implications for clinicians and medical policy	9
Model performance	16	Report performance measures (with CIs) for the prediction model.	R: Overall predictive performance	4
Model-updating	17	If done, report the results from any model updating (i.e., model specification, model performance).	M: Model updating; R: Overall predictive performance	18, 4
**Discussion**				
Limitations	18	Discuss any limitations of the study (such as nonrepresentative sample, few events per predictor, missing data).	D: Implications for clinicians and medical policy	9

Interpretation	19a	For validation, discuss the results with reference to performance in the development data, and any other validation data.	D: Relation to other studies	9
	19b	Give an overall interpretation of the results, considering objectives, limitations, results from similar studies, and other relevant evidence.	D: Relation to other studies	9
Implications	20	Discuss the potential clinical use of the model and implications for future research.	D: Implications for clinicians and medical policy	9
Other information				
Supplementary information	21	Provide information about the availability of supplementary resources, such as study protocol, Web calculator, and data sets.	Supplementary material index	S2
Funding	22	Give the source of funding and the role of the funders for the present study.	Acknowledgements	19

Supplementary Table 1. TRIPOD guidelines and pages where discussed [2]

Type	Name	Description	Records	Individuals	End of pre-prediction (before start of risk year)
Raw EHR	PIS	GP prescribing information	393,573,549	5,589,772	One month based upon the Paid Date
	SMR00	Outpatient attendances	27,463,987	3,753,240	One day based upon the Clinic Date
	SMR01	Acute inpatients and day cases	26,326,889	2,205,606	One day based upon the Date of Admission
	SystemWatch	Urgent care monitoring	12,890,591	1,750,009	One day based upon the Date of Admission
	A&E2	Accident and emergency records	7,539,454	3,031,773	One day based upon the Date of Arrival
	SMR01E	Geriatric long stay	137,340	25,015	.
	SMR04	Mental health inpatient and day cases	111,487	51,635	One day based upon the Date of Admission
	**All raw EHR tables combined**		468,043,297	5,829,532	
Other	SPARRALTC	Long-term conditions	3,286,987	1,978,171	.
	Deaths	Mortality records	283,554	283,554	

Supplementary Table 2. Input data sources. The data comprises national EHR Scottish databases between 1 May 2013 and 30 April 2018. Each record corresponds to a single interaction with the health system. SMR - Scottish Morbidity Records. Information about specific EHR tables is available at [3] (SMR datasets), [4] (A&E2), [5] (System Watch) and [6] (PIS). The LTC table was derived by PHS from historic SMR01 tables with an admission between 01 January 1981 and 30 April 2018. The Deaths table was provided to PHS by National Records of Scotland (NRS) and is up to date until TBC. The timescale for the pre-prediction period is 3 years for PIS, SMR00, SMR01, System Watch, A&E2, SMR01E and SMR04. All LTC records up to 01 January 1981 are used in the pre-prediction period. The earlier end of the pre-prediction period is because the most recent month will not be generally available when running the predictions.

Class	Variable name	Name
Analysis only	time	Time cutoff
	target	Emergency admission in year following cutoff date
	reason	Whether admitted or died or both
	cv	Fold for cross-validation
Demographics	sexM	Male sex
	age	Age at time cutoff
	SIMD_DECILE_2016_SCT	SIMD decile
General	v3score	SPARRA version 3 score
Previous admissions	emergency_bed_days	Number of emergency bed days
	other_bed_days	Number of non emergency non elective bed days
	num_emergency_admissions	Number of emergency admissions
	elective_bed_days	Number of elective bed days
	num_elective_admissions	Number of elective admissions
	num_emergency_selfharm	Number of self harm related emergency admissions
	num_other_admissions	Number of non emergency non elective admissions
	num_alcohol_substance_admissions	Number of emergency drug and alcohol-related admissions
	num_alcohol_admissions	Number of emergency alcohol-related admissions
	numLTCs_resulting_in_admin	Number of long-term conditions resulting in admission
	numLTCs_resulting_in_elective_admin	Number of long-term conditions resulting in elective admission
	numLTCs_resulting_in_emergency_admin	Number of long-term conditions resulting in emergency admission
	numLTCs_resulting_in_other_admin	Number of long-term conditions resulting in non-emergency non-elective admission
	num_psych_admissions	Number of previous psychiatric admissions
	num_dc_admissions	Number of previous day case admissions
	days_since_last_acute	Days since last acute admission of any type
	days_since_last_emergency_admission	Days since last emergency admission

	days_since_last_elective_admission	Days since last elective admission
A&E attendances	num_ae2_attendances	Number of previous A&E attendances
	num_alcohol_drug_attendances	Number of drug and alcohol related A&E attendances
	num_psych_attendances	Number of psychiatric A&E attendances
	days_since_last_AE2	Days since last A&E attendance
Prescriptions	num_bnf_sections	Number of BNF sections from which a prescription was filled
	num_bnf_total	Total number of filled prescriptions
	pis_respiratory	Number of respiratory-related prescriptions
	pis_corticosteroids	Number of corticosteroid prescriptions
	pis_antianaemics	Number of anti-anaemic prescriptions
	pis_cns	Number of central nervous system related prescriptions
	pis_bronco	Number of bronchodilator prescriptions
	pis_infections	Number of infection related prescriptions
	pis_endocrine	Number of endocrine-related prescriptions
	pis_incontinence	Number of incontinence device presciptions
	pis_stoma	Number of stoma device prescriptions
	pis_anticoagulant	Number of anticoagulant and protamine prescriptions
	pis_antibacterial	Number of antibacterial prescriptions
	pis_antiepileptic_Drugs	Number of antiepileptic prescriptions
	pis_antifibrinolytic	Number of antifibrinolytic and haemostatic prescriptions
	pis_antisecretory	Number of antisecretory and mucosal protectant prescriptions
	pis_gut_motility	Number of antispasmodic and gut-motility altering prescriptions
	pis_diuretics	Number of diuretic prescriptions

pis_lipid	Number of lipid-lowering drug prescriptions
pis_bandages	Number of arm sling bandage prescriptions
pis_catheters	Number of catheter prescriptions
pis_antiplatelets	Number of antiplatelet prescriptions
pis_respiratory_corticosteroids	Number of respiratory corticosteroid prescriptions
pis_dementia	Number of dementia-related prescriptions
pis_skin	Number of skin condition prescriptions
pis_hypertensive_heart_failure	Number of hypertensive heart failure prescriptions
pis_intestinal	Number of prescriptions for drugs affecting intestinal secretions
pis_diabetes	Number of prescriptions for drugs used in diabetes mellitus
pis_neuromuscular	Number of prescriptions for drugs used in neuromuscular disorders
pis_parkinsonism	Number of prescriptions for drugs used in Parkinsonism and related disorders
pis_sub_depend	Number of prescriptions for drugs used in substance dependence
pis_fluids	Number of fluid and electrolyte prescriptions
pis_Minerals	Number of mineral prescriptions
pis_mucolytics	Number of mucolytic prescriptions
pis_nose	Number of drugs acting on the nose prescriptions
pis_gastro_int	Number of gastrointestinal drug prescriptions
pis_betablockers	Number of beta-blocker prescriptions
pis_antianginal	Number of antianginal prescriptions
pis_nutrition	Number of oral nutrition prescriptions

	pis_sex_hormone_antagonists	Number of sex hormone antagonists prescriptions
	pis_genitourinary	Number of genitourinary prescriptions
	pis_inotropic	Number of inotropic prescriptions
	pis_ear	Number of ear prescriptions
	pis_glaucoma	Number of glaucoma prescriptions
	pis_antibacterial_eyes	Number of eye antibacterial prescriptions
	pis_ophthalmic	Number of ophthalmic prescriptions
	pis_antiinflammatory_corticosteroids	Number of antiinflammatory corticosteroid prescriptions
	pis_cromo	Number of cromoglycate prescriptions
	pis_oropharynx	Number of oropharyngeal prescriptions
	pis_antiarrhythmics	Number of antiarrhythmic prescriptions
	pis_immune	Number of immunomodulatory prescriptions
	pis_hosiery	Number of hosiery prescriptions
	pis_cytotoxics	Number of cytotoxics prescriptions
	pis_supplements	Number of supplement prescriptions
	pis_mydriatics	Number of mydriatics prescriptions
	pis_food	Number of food prescriptions
	pis_metabolic	Number of metabolic prescriptions
	pis_local_anaesthetics	Number of local anaesthetic prescriptions
	pis_rheumatic	Number of rheumatic disease prescriptions
	pis_vitamins	Number of vitamin prescriptions
Long-term conditions	num_general_ltc	Number of recorded long term conditions
	parkinsons_indicated	Parkinsons disease indicated by admission records or medication
	diabetes_indicated	Diabetes indicated by admission records or medication

MS_indicated	Multiple sclerosis indicated by admission records or medication
epilepsy_indicated	Epilepsy indicated by admission records or medication
dementia_indicated	Dementia indicated by admission records or medication
CONGENITAL_PROBLEMS	Previous congenital problems diagnosis
ENDOCRINE_MET	Previous endocrine or metabolic disease diagnosis
DIS_BLOOD	Previous blood disease diagnosis
ARTHRITIS	Previous arthritis diagnosis
OTHER_DIGESTIVE	Previous digestive disorder diagnosis
ASTHMA	Previous asthma diagnosis
ATRIAL_FIBRILLATION	Previous atrial fibrillation diagnosis
CANCER	Previous cancer diagnosis
CHRONIC_LIVER_DISEASE	Previous chronic liver disease diagnosis
COPD	Previous chronic obstructive pulmonary disease diagnosis
DEMENTIA	Previous dementia diagnosis
DIABETES	Previous diabetes mellitus diagnosis
EPILEPSY	Previous epilepsy diagnosis
HEART_DISEASE	Previous heart disease diagnosis
HEART_FAILURE	Previous heart failure diagnosis
MULTIPLE_SCLEROSIS	Previous multiple sclerosis diagnosis
PARKINSON_DISEASE	Previous Parkinsons disease diagnosis
RENAL_FAILURE	Previous renal failure diagnosis
CEREBROVASCULAR_DISEASE	Previous cerebrovascular disease diagnosis
ltc_FIRST_ARTHRITIS_EPISODE_yearssincediag	Years since first arthritis diagnosis
ltc_FIRST_CONGENITAL_PROBLEMS_EPISODE_yearssincediag	Years since first congenital problems diagnosis
ltc_FIRST_ENDOCRINE_MET_EPISODE_yearssincediag	Years since first endocrine or metabolic disease diagnosis
ltc_FIRST_DIS_BLOOD_EPISODE_yearssincediag	Years since first blood disease diagnosis
ltc_FIRST_OTHER_DIGESTIVE_EPISODE_yearssincediag	Years since first digestive disorder diagnosis

	ltc_FIRST_ASTHMA_EPISODE_yearssincediag	Years since first asthma diagnosis
	ltc_FIRST_ATRIAL_FIBRILLATION_EPISODE_yearssincediag	Years since first atrial fibrillation diagnosis
	ltc_FIRST_CANCER_EPISODE_yearssincediag	Years since first cancer diagnosis
	ltc_FIRST_CHRONIC_LIVER_DISEASE_EPISODE_yearssincediag	Years since first chronic liver disease diagnosis
	ltc_FIRST_COPD_EPISODE_yearssincediag	Years since first chronic obstructive pulmonary disease diagnosis
	ltc_FIRST_DEMENTIA_EPISODE_yearssincediag	Years since first dementia diagnosis
	ltc_FIRST_DIABETES_EPISODE_yearssincediag	Years since first diabetes mellitus diagnosis
	ltc_FIRST_EPILEPSY_EPISODE_yearssincediag	Years since first epilepsy diagnosis
	ltc_FIRST_HEART_DISEASE_EPISODE_yearssincediag	Years since first heart disease diagnosis
	ltc_FIRST_HEART_FAILURE_EPISODE_yearssincediag	Years since first heart failure diagnosis
	ltc_FIRST_MULTIPLE_SCLEROSIS_EPISODE_yearssincediag	Years since first multiple sclerosis diagnosis
	ltc_FIRST_PARKINSON_DISEASE_EPISODE_yearssincediag	Years since first Parkinsons disease diagnosis
	ltc_FIRST_RENAL_FAILURE_EPISODE_yearssincediag	Years since first renal failure diagnosis
	ltc_FIRST_CEREBROVASCULAR_DISEASE_EPISODE_yearssincediag	Years since first cerebrovascular disease diagnosis
	ltc_total_count	Number of recorded long term conditions
Outpatients	num_outpatient_appointment_general	Number of previous first outpatient appointments
	num_outpatient_appointment_psych	Number of previous first psychiatric outpatient appointments
	num_outpatient_appointment_followup_psych	Number of previous follow-up psychiatric outpatient appointments
	num_outpatient_appointment_followup_general	Number of follow-up outpatient appointments
	days_since_last_SMR00	Days since last outpatient attendance
	days_since_last_SMR04	Days since last mental health and day case attendance

Supplementary Table 3. Definition of input features for SPARRAv4. Variable names match the names used in our analysis code.

Words	Label
Antihist, Hyposensit & Allergic Emergen<br>Drugs Acting On The Nose<br>Corti'roids & Other Anti-Inflamm.Preps.<br>Nasal Products	Nasal allergies
Contraceptives<br>Miscellaneous Ophthalmic Preparations<br>Eye Products<br>Antiviral Drugs<br>Corti'roids & Other Anti-Inflamm.Preps.<br>Anti-Infective Eye Preparations	Eye products
Antidepressant Drugs<br>Antibacterial Drugs	
(BNF) Unknown	
Wound Management & Other Dressings<br>Drugs Used In Neuromuscular Disorders<br>Antibacterial Drugs<br>Skin Fillers And Protectives<br>Night Drainage Bags<br>Catheters<br>Leg Bags<br>Stockinette<br>Arm Sling/Bandages<br>Surgical Adhesive Tape<br>Swabs<br>Irrigation Solutions<br>*Urinary tract infection, site not specified*<br>*Essential (primary) hypertension*	Wound management
Hypnotics And Anxiolytics<br>Drugs Used In Substance Dependence	Substance Abuse and Mental Health
Acne and Rosacea<br>Sex Hormones & Antag In Malig Disease<br>Hypothalamic&Pituitary Hormones&Antioest<br>Antibacterial Drugs<br>*Chemotherapy session for neoplasm*<br>*Malignant neoplasm, breast, unspecified*	
Antiplatelet Drugs<br>Lipid-Regulating Drugs	Metabolic syndrome
Oral Nutrition<br>Preparations For Warts And Calluses<br>Top Local Anaesthetics & Antipruritics<br>Vaccines And Antisera<br>Anti-Infective Skin Preparations<br>Other Appliances<br>Anthelmintics<br>Anti-Infective Eye Preparations<br>Cough Preparations<br>Drugs Acting On The Oropharynx<br>Antiperspirants<br>Base/Dil/Susp Agents/Stabilisers<br>*Other chemotherapy*<br>*Viral infection, unspecified*	Skin disease

Thyroid And Antithyroid Drugs<br>CNS Stimulants and drugs used for ADHD<br>Antibacterial Drugs	
Analgesics	
Antibacterial Drugs<br>Anti-Infective Skin Preparations<br>Drugs Acting On The Oropharynx<br>Treatment Of Vaginal & Vulval Conditions<br>Antifungal Drugs<br>Drugs Acting On The Ear<br>Topical Corticosteroids<br>Cough Preparations<br>Anti-Infective Eye Preparations	
Hypertension and Heart Failure	
Anaemias + Other Blood Disorders<br>Chronic Bowel Disorders<br>Antifibrinolytic Drugs & Haemostatics<br>Cytotoxic Drugs	
Dyspep&Gastro-Oesophageal Reflux Disease<br>Drugs Used In Psychoses & Rel.Disorders<br>Drugs Used In Park'ism/Related Disorders<br>Acute Diarrhoea<br>Ileostomy Bags<br>Adhesive Removers (Sprays/Liquids/Wipes)<br>Colostomy Bags<br>Swabs<br>Skin Fillers And Protectives	Stoma care
Emollient & Barrier Preparations<br>Topical Corticosteroids<br>Emollients<br>Shampoo&Other Preps For Scalp&Hair Cond<br>Preparations For Eczema And Psoriasis<br>Anti-Infective Skin Preparations	Skin and scalp disorders
Corticosteroids (Endocrine)<br>Drugs Affecting The Immune Response<br>Fluids And Electrolytes<br>Minerals<br>Sunscreens And Camouflagers<br>Antibacterial Drugs<br>Acute Diarrhoea	
Antisecretory Drugs+Mucosal Protectants<br>Antibacterial Drugs	
Lipid-Regulating Drugs	
Beta-Adrenoceptor Blocking Drugs	
Drugs For Genito-Urinary Disorders<br>Sex Hormones	

ICD10 code begins with:	Admission type
A;B	Infectious disease
C	Neoplasm
D1;D2;D3;D4	Neoplasm
D5;D6;D7;D8;D9	Blood
E	Endocrine/metabolic
F	Mental/behavioural
G	Nervous system
H1;H2;H3;H4;H5	Eye
H6;H7;H8;H9	Ear
I	Circulatory
J	Respiratory
K	Digestive
L	Skin
M	Musculoskeletal
N	Genitourinary
O	Obstetric/puerperium
P	Perinatal
Q	Congenital
R	Abnormality NEC
S;T;V;X;Y	External
U;Z	Other

Supplementary Table 5. Definition of different admission types.

Antibacterial Drugs	
Anticoagulants And Protamine<br>Treatment Of Glaucoma<br>Positive Inotropic Drugs<br>Anti-Arrhythmic Drugs<br>*Atrial fibrillation and flutter*	Arrhythmias
Nit,Calc Block & Other Antianginal Drugs	
Soft-Tissue Disorders & Topical Pain Rel<br>Local Anaesthesia<br>Analgesics<br>Elastic Hosiery<br>Dry Mouth Products<br>Acute Diarrhoea	

Supplementary Table 4. Exploration of the inferred topics. Details of derived topics for topic model used for prediction in F1 (fitted to F2+F3). A topic model assumes that each 'document' (individual) in a 'corpus' (population) is associated with various 'topics' (roughly, illness categories) where each topic corresponds to a distribution over 'words' (ICD10 codes and medication types). We would expect that the 30 topics fitted to each fold roughly represent the major clusters of disease types which occur amongst those individuals. This tables shows the 'words' with the highest probability of membership in each topic ($> 1\%$, where probabilities over all words sum to 100%). In each topic, words are ordered by decreasing probability of topic membership. ICD10 codes are italicised; medication types are not. Topics are ordered by decreasing importance (mean absolute Shapley value). We manually assigned labels to some topics which appear to code for particular disease types.

	Total	Admitted only	Died	Both
Excluded	4,622,512	128,241	6,139	3,166
Included	12,866,084	977,159	57,183	107,827
Died	585,322	0	0	0
No v3	4,528,514	121,857	6,036	3,011
No SIMD	199,746	12,688	151	263
Unmatched	13,524	526	0	0
Died and No v3	585,089	0	0	0
Died and No SIMD	1,258	0	0	0
Died and Unmatched	0	0	0	0
No v3 and No SIMD	112,992	6,648	48	110
No v3 and Unmatched	6,468	179	0	0
No SIMD and Unmatched	303	20	0	0
Died and No v3 and No SIMD	1,258	0	0	0
Died and No v3 and Unmatched	0	0	0	0
Died and No SIMD and Unmatched	0	0	0	0
No v3 and No SIMD and Unmatched	258	17	0	0
All	0	0	0	0

Supplementary Table 6. Frequency of EA and deaths amongst samples excluded from SPARRAv4. All figures show total numbers. In row names, as for Figure 1b: 'Died': Died prior to time cutoff; 'No SIMD': missing SIMD; 'No v3': missing SPARRAv3 score; 'Unmatched': could not match record. For completeness, combinations of exclusions are included even if no individual was excluded with this particular combination.

	Fold 1			
	YED	U16	FEC	LTC
ANN	0	0	0	0
Penalised GLM	0	0	0	0
Naive Bayes	0	0	0	0
RF, depth: 20	0	0	0.24	0.07
RF, depth: 40	0.18	0.21	0.17	0.23
SPARRAv3	0	0	0	0
XGB depth:3	1.86	1.57	1.24	1.69
XGB depth:4	0.84	1.57	0.68	0.62
XGB depth: 8	3.12	3.20	2.14	2.39

	Fold 2			
	YED	U16	FEC	LTC
ANN	0	0	0	0
Penalised GLM	0	0	0	0
Naive Bayes	0	0	0	0
RF, depth: 20	0	0	0.09	0.01
RF, depth: 40	0.22	0.20	0.24	0.16
SPARRAv3	0	0	0	0
XGB depth:3	1.87	1.58	1.37	1.62
XGB depth:4	0.88	1.72	0.68	0.74
XGB depth: 8	3.02	3.45	2.07	2.47

	Fold 3			
	YED	U16	FEC	LTC
ANN	0	0	0	0
Penalised GLM	0	0	0	0
Naive Bayes	0	0	0	0
RF, depth: 20	0	0	0.28	0
RF, depth: 40	0.16	0.11	0.15	0
SPARRAv3	0	0	0	0
XGB depth:3	1.73	1.16	1.31	1.60
XGB depth:4	1.07	1.82	0.67	0.48
XGB depth: 8	3.02	3.42	2.06	2.44

Supplementary Table 7. Coefficients of ensemble when fitted separately to SPARRA*v*3 cohorts. Columns YED, U16, FEC, and LTC correspond to subcohorts in SPARRA*v*3; please see Methods.

SUPPLEMENTARY FIGURES

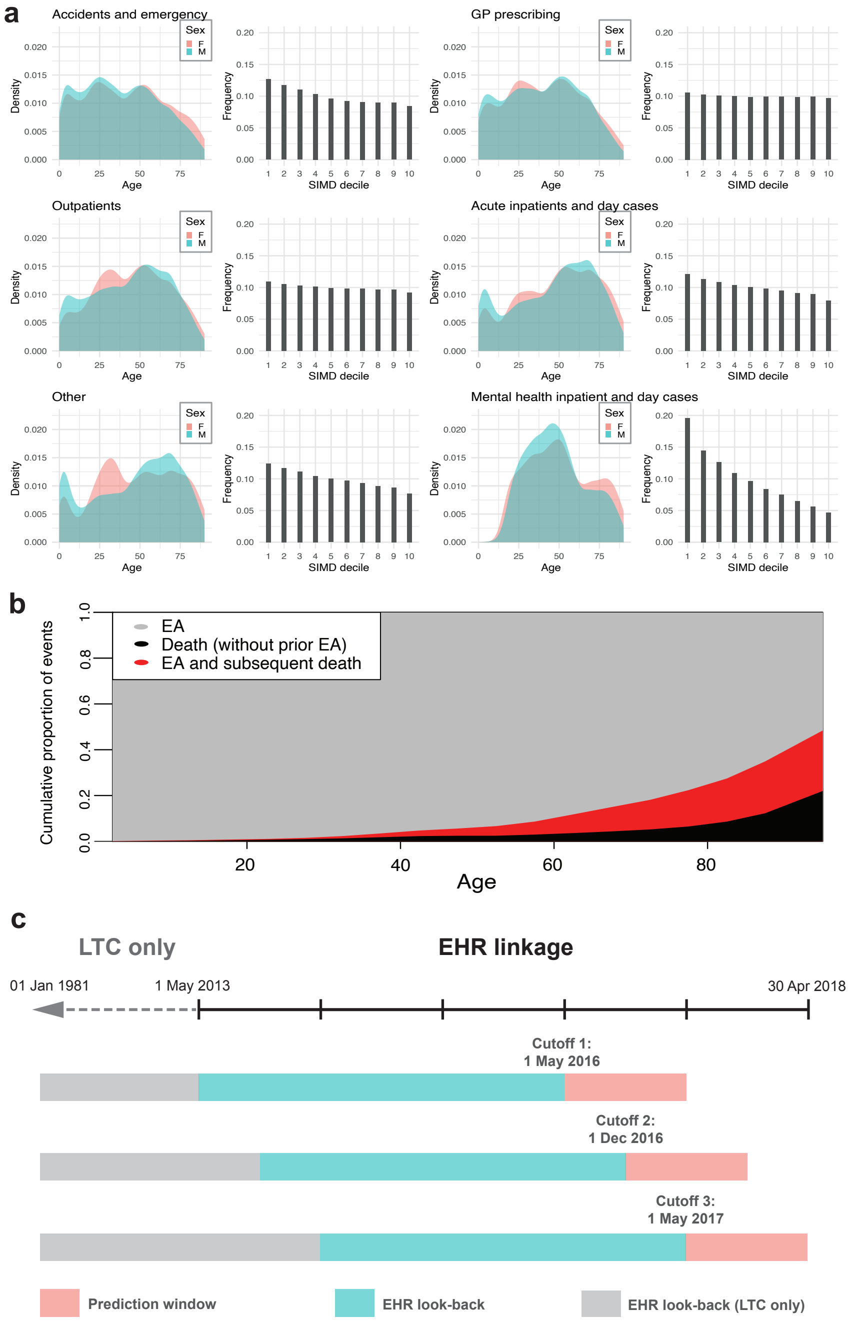

Supplementary Figure 1. Extended data overview. (A) Distribution of the number of input EHR entries (prior to exclusions) according to age, sex and SIMD deciles (1: most deprived; 10: least deprived) stratified by the input database. All sub-panels are drawn to the same scale. “Other” includes geriatric long stay (SMR01E) and urgent care monitoring (System Watch). (B) Distribution of target events by age stratified by event type. (C) Time cutoffs used for defining prediction periods and look-back times.

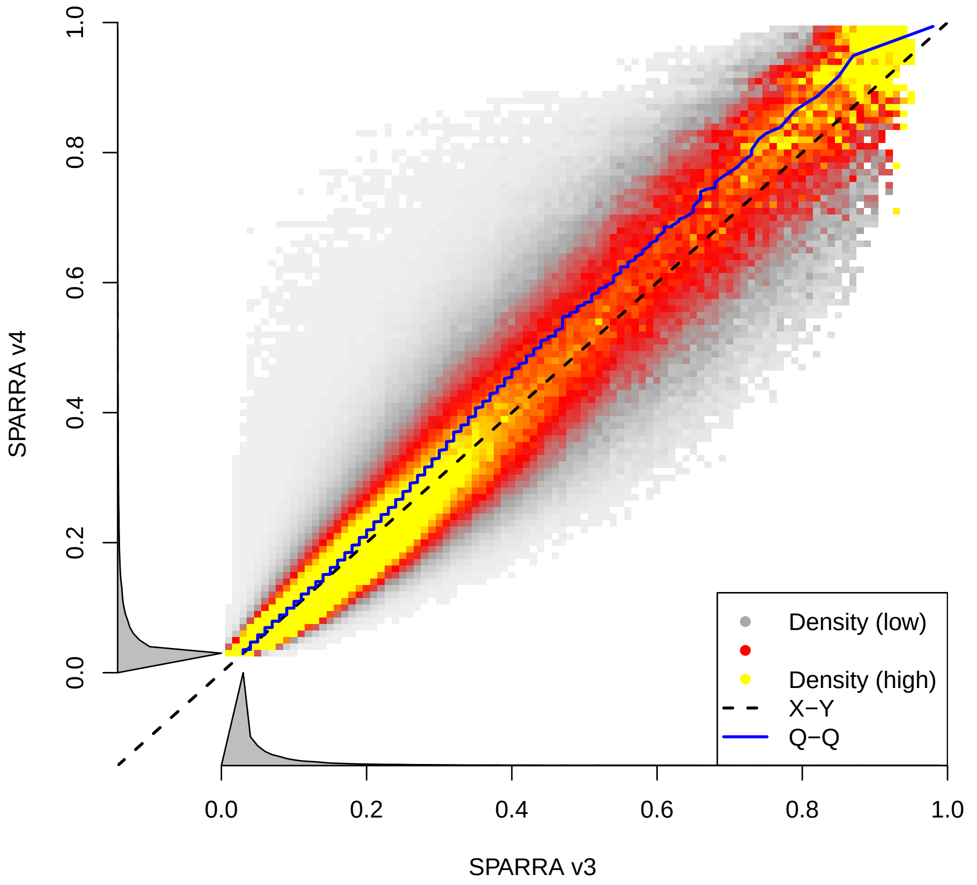

Supplementary Figure 2. Density plot comparing SPARRAv3 and SPARRAv4 scores. The test datasets used within each CV iteration were combined in order to generate this plot (i.e. all samples are included once). Joint density (low to high: white-grey-red-yellow) of individual SPARRAv3 and SPARRAv4 scores. The density is normalised to uniform marginal on the Y axis, then the X axis; true marginal distributions of risk scores are shown alongside in grey.

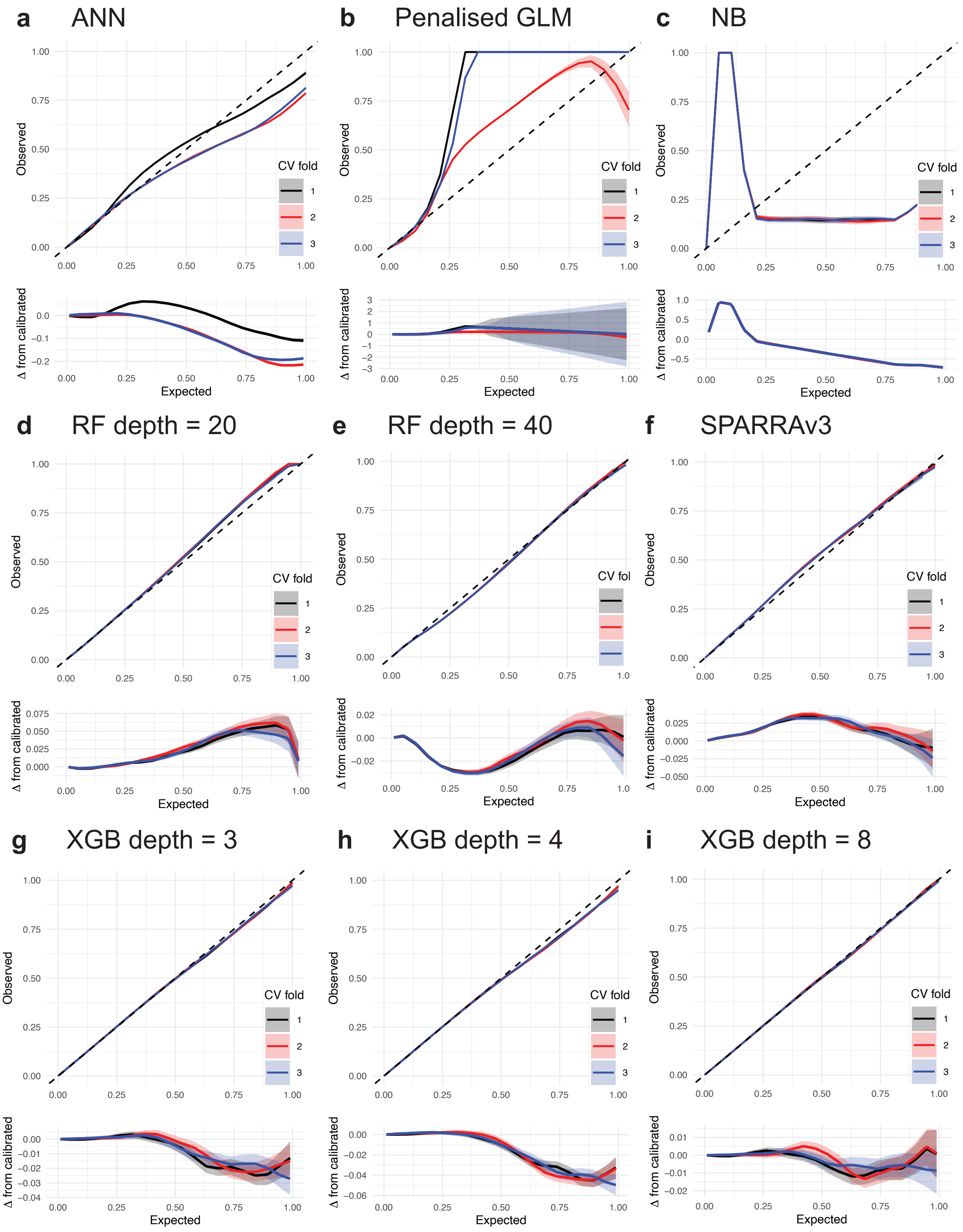

Supplementary Figure 3. Calibration curves for SPARRAv4 model constituents. (A-I) Estimates obtained for the test set within each CV iteration are shown in different colours (legend shown in top left panel). Bottom sub-panels show departure from perfect calibration.

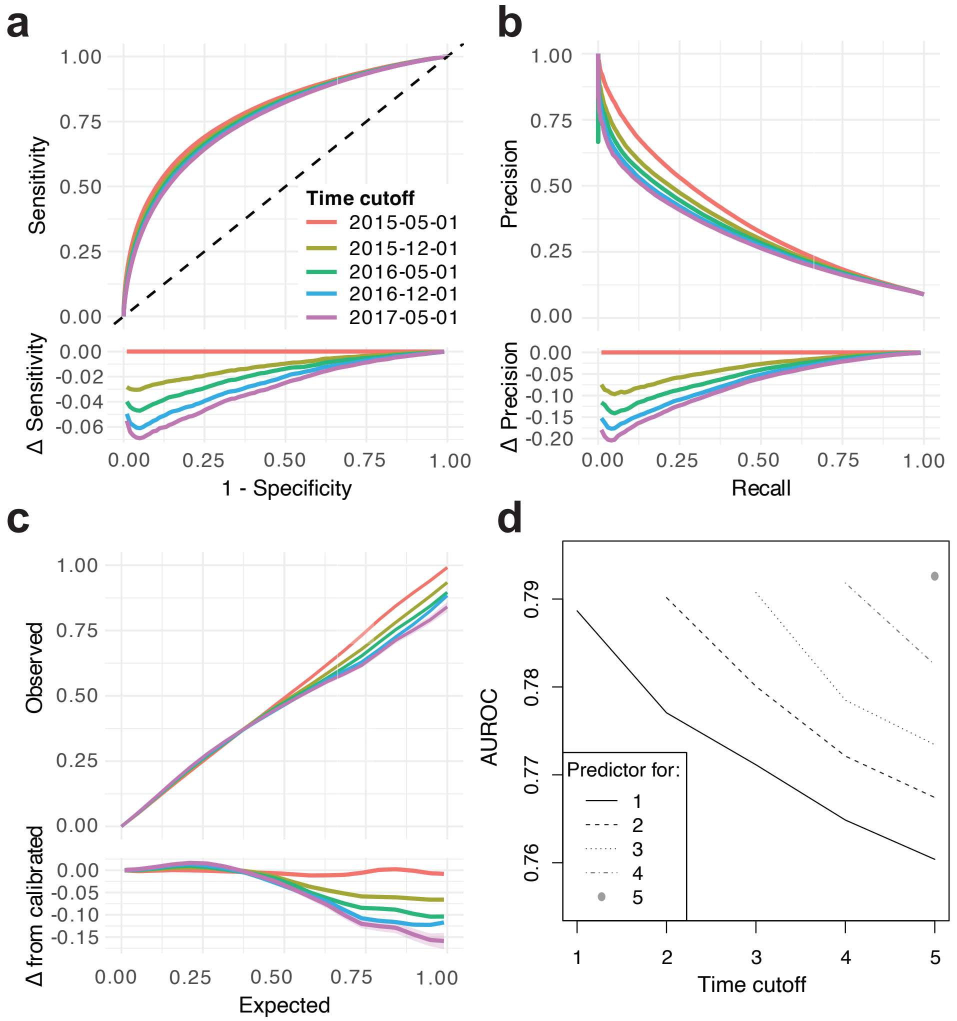

Supplementary Figure 4. Performance of a static model and static scores used to predict risk at future time cutoffs. (A-C) Performance of static scores evaluated using M_0 at time t_0 for predicting EA at times $t_1 - t_5$. (A) ROC curves. (B) PR curves. (C) Calibration curves. (D) AUROCs for scores calculated at each time cutoff (based on M_0) for prediction in subsequent time cutoffs.

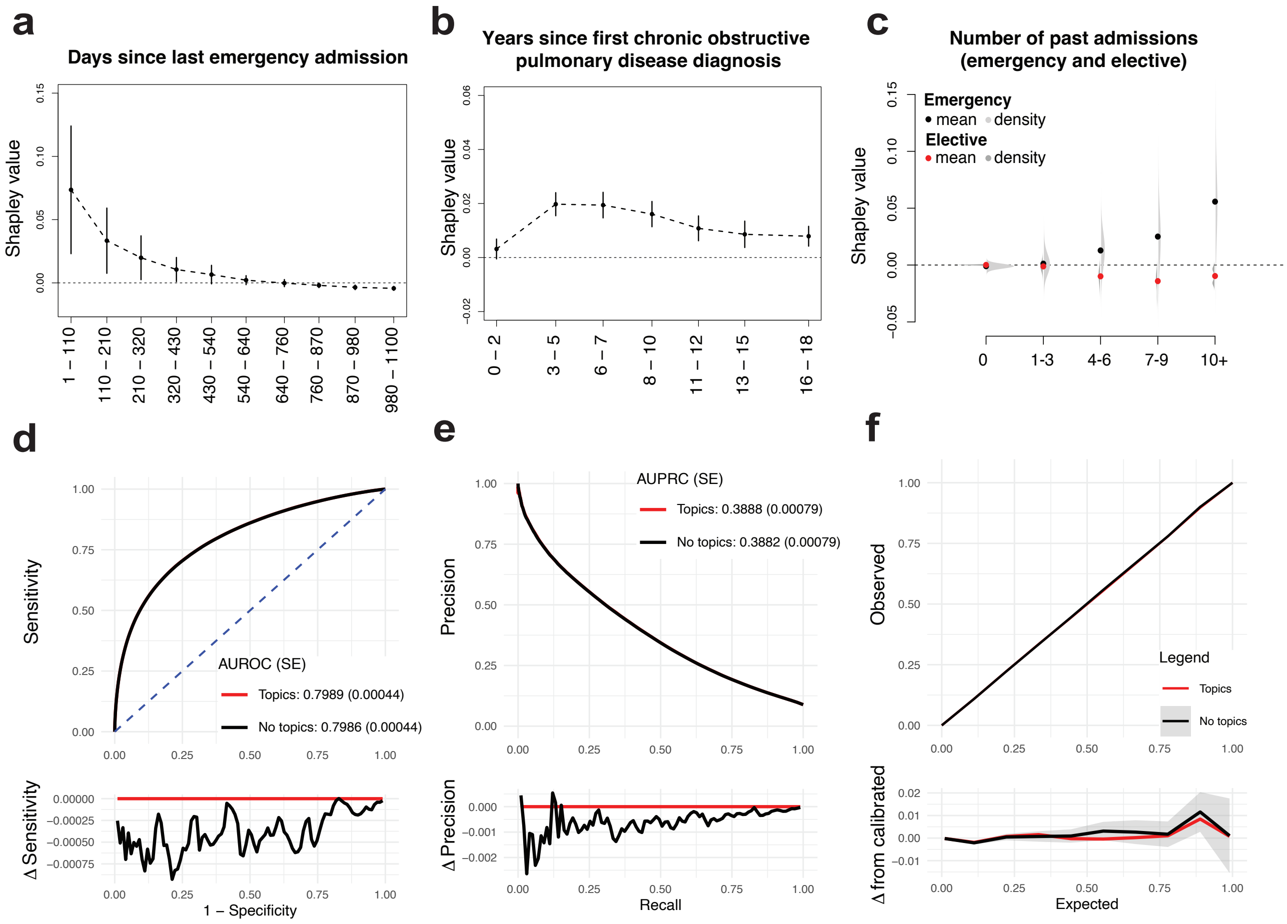

Supplementary Figure 5. Feature importance. (A-B) Two examples of non-linear feature importance as measured by mean Shapley values (vertical lines show plus/minus one standard deviation). (C) Distribution of Shapley values for the number of previous elective and emergency admissions. (D-E) Comparison of predictive performance with and without topic-model derived features. (D) ROC. (E) PRC. (F) Calibration curve. For (D-E), bottom sub-panels show differences in sensitivity and specificity, respectively. In (F) bottom sub-panel shows difference with respect to perfect calibration.

SUPPLEMENTARY NOTES

1 Analysis of specific topic effects

We searched for individuals for whom the topic model made a substantial difference to their SPARRA score. We considered Shapley values for the 30 topic features across 20,000 uniformly-randomly-chosen individuals in cross-validation fold 1. We searched for topic-derived features for which some of these 20,000 individuals had a Shapley value in excess of 2% for that topic. This is a large Shapley value; for reference, the mean Shapley value from being in the most-deprived decile is only around 1%.

We noted that for one topic feature (topic 21) 87 individuals (about 0.43% of the cohort of 20,000) had a Shapley value in excess of 2%, meaning that the additive contribution to their score from the topic feature was at least 0.02. To gauge the effect of this change, we compared the SPARRAv3 and SPARRAv4 scores of these individuals (the SPARRAv3 score does not use features derived from a topic model). The SPARRAv4 scores were on average higher (mean 0.52 for SPARRAv4 vs 0.40 for SPARRAv3; p-value (t-test) $< 1 \times 10^{-4}$) and calibration-in-the-large was closer for SPARRAv4 (admission frequency 0.56; closer to 0.52 (SPARRAv4) than 0.40 (SPARRAv3)).

Topic 21 was associated with skin and scalp disorders (see Supplementary Table 4). It is possible that the individiuals for whom this topic feature had a high Shapley value were at elevated risk of EA due to such disorders, but that this was not detectable from the features used in SPARRAv3.

In order to avoid data leakage while using our cross-validation scheme, we needed to fit three separate topic models, each fitted to data from two cross-validation folds and used to generate topic features for the remaining fold (see Methods section). For the deployed model, a topic model is refitted to the entire cohort, so the inferred topics are unlikely to contain the exact same cluster of prescriptions and diagnosis. Furthermore, topic-specific contributions may differ from those presented here. However, the analysis above does indicate that, in general, topic features can lead to substantial improvements in score accuracy for some individuals.

2 Model re-calibration

We applied a monotonic transformation to optimise the calibration of the scores generated by the ensemble. Given a predicted value $\tilde{Y}$ (for ease of notation we do not explicitly include its dependency on the input features X) we defined a transformation $m(\cdot)$ to optimise calibration, essentially using isotonic regression. The latter was derived using the following procedure.

Fitst, we defined an empirical calibration function for an estimator $\tilde{Y}$ of $Y|X$:

$$\begin{aligned} CAL_{\tilde{Y}}(y) &= \text{mean}\left(\tilde{Y}\middle|\left(|\tilde{Y}-y| < \frac{1}{100}\right)\right) \\ &\approx \mathbb{E}_{Y|X}\left(Y|\tilde{Y}=y\right) \end{aligned} \tag{1}$$

We then found a, b such that the mean and mode of $(a\tilde{Y}+b)$ were approximately correctly calibrated; that is, $CAL_{a\tilde{Y}+b}(y) = y$ for $y \in \{\text{mean}(a\tilde{Y}+b), \text{mode}(a\tilde{Y}+b)\}$, and scaled a, b such that $0 \leq a\tilde{Y}+b \leq 1$. Across an evenly spaced grid G of 100 y-values we computed the function:

$$c(y) = (1-10^{-5}) \max_{y' \in G; y' \leq y} CAL_{a\tilde{Y}+b}(y') + 10^{-5}y \tag{2}$$

using the cumulative maximum of $CAL(\cdot)$ to ensure $c(\cdot)$ is non-decreasing, and adding a linear term to ensure $c(\cdot)$ is increasing. We extended the domain of $c(\cdot)$ to $[0,1]$ using piecewise linear interpolation, and defined our calibrating transform $m(\cdot)$ as the inverse of $c(\cdot)$:

$$m(y) = c^{-1}(ay+b) \tag{3}$$

The transformation above was optimised using by further splitting the training set (F1+F2) within our 3-fold cross-validation (CV) procedure (we use F1, F2 and F3 to denote each fold). For each CV-fold, the following steps were performed:

1. Train all constituent models using F1 (except SPARRAv3, for which PHS provided the scores).

2. Each constituent model was then used to generate scores for samples in F2.

3. Given those scores, ensemble weights were inferred via 10-fold CV within F2.

4. Using the previously calculated scores and ensemble weights, the parameters a and b were chosen to optimise calibration in F2.

5. The optimal ensemble weights and calibration transformation parameters (a and b) were then used as fixed constants when training the model in the combined F1+F2 dataset.

Note that, due to computational constrains, the topic model was not retrained within the above procedure. Instead, a pre-trained topic model (using F1+F2 as a combined dataset) was used to generate features to be used in step 1.

3 Use of subcohort-specific ensemble coefficients

When fitting the SPARRAv4 score, for each cross-validation fold, we computed predictions for a range of constituent machine learning models. The final score was defined as a linear combination of the predictions generated by each constituent model a(see Methods section and Table 2). The optimal linear combination was determined by fitting an L-1 penalised generalised linear model with the predictions from the constituent models as input and presence of EA as output.

We considered the possibility that the model could be improved by allowing the coefficients of each model to vary across different subsets of the population. In particular, we assessed the extent to which allowing models to vary across the subcohorts used in the SPARRAv3 score (LTC, YED, FEC, and U16; see Methods section) improved the overall performance of the model. We fitted separate penalised regression models (leading to different ensemble weights) in each of the three cross-validation folds and four cohorts (for 12 overall). Subsequently, we assessed how predictive performance changed in the corresponding subcohort in the test set. For instance, we fit one linear model to samples in the YED cohort in folds 2 and 3, and evaluated the performance of this model in samples in the YED cohort in fold 1.

For comparison, we also considered the performance of our original non-cohort-specific ensemble weights, the performance of the best constituent model, and the performance of our original model without topic-model derived features. We evaluated all models using AUROC.

We found that using cohort-specific coefficients in this way improved AUC in the relevant test sets (of 12 comparisons of AUROC, 11 improved; $p < 0.007$ using a Binomial test). However, the magnitude of the change was small: AUC improved in each subcohort by a mean value of only 3.8×10^{-5} (where the mean is across cross-validation folds and cohorts). By comparison, use of topic features improves AUC by a mean of 4.1×10^{-4}, around ten times higher, and use of a weighted sum of models rather than just the best-performing constituent model improves AUC by 7.7×10^{-4}, around twenty times higher.

Given the relatively small advantage of doing this relative to the difficulty of implementation, we opted not to fit separate models in subcohorts in this way. However, this remains an active area of further research.

Supplementary Table 7 details the coefficients attained in each model. Generally, the same models (XGB and RF) had non-zero coefficients in each case.

4 Imputation of lengths of stay when discharge date was missing

Some of our predictors concerned lengths-of-stay; that is, total days spent in hospital in the pre-prediction period (`elective_bed_days`, `emergency_bed_days`, and `other_bed_days`; see Supplementary Table 3). In general, these were calculated by finding all stays listed for a given individual, subtracting the admission date from the discharge date for each stay, and summing the results across all stays. However, for some hospital stays, no discharge date was present in the source tables. In some cases, this was due to the individual still being in hospital at the time cutoff, but in others was evidently due to the discharge date simply not being recorded; we identified several individuals who were admitted with no discharge date who had evidence of community activity during the time they were supposedly in the hospital. To manage this, we used an imputation procedure for hospital stays in which the discharge date was not recorded. When we see an individual at a time cutoff t with admission date d and no discharge date, we have options of:

1. Do not count this admission towards the total length of stays; that is, count the stay length as 0 days for that admission. This will under-estimate the total length of stay.

2. Count time $t-d$ towards the total length of stay. Effectively this imputes the discharge date using the time cutoff. This could lead to incorrect assumptions of very long hospital stays for individuals; indeed, since the pre-prediction period is three years, the mean assumed hospital stay length for such patients would be in excess of eighteen months. This is likely to over-estimate the total length of stay.

3. Count some arbitrary time t_0 towards the total length of stay. Depending on the value of t_0, the total length of stay may be under- or over-estimated.

All of these options coult potentially decrease the usefulness of these variables by artificially inflating (or deflating) the predicted EA risk. As a compromise, we decided to use

$$\min(t-d, t_0) \tag{4}$$

as the length of stay for admissions with a missing discharge date. Effectively, this strategy uses t_0 as a default *minimum length* for stays with missing discharge date.

To choose t_0, we use an empirical Bayes-optimal decision rule. Let E be the event that the discharge time for a given admission is not recorded. We model the time $t-d$ as a (discrete) random variable X with a mixture distribution depending on E. We want to choose t_0 so that $P(E|X=x) \geq 1/2$ if and only if $x \geq t_0$. We set

$$P(X=x|\neg E) = f(x)$$
$$P(X=x|E) = \frac{1}{3 \times 365.25} = c$$

that is; if the discharge time is recorded (in which case the individual is genuinely still in hospital at time t), we have some distribution of true lengths of stay, whereas if the discharge time is not recorded, the time $t-d$ has an equal probability of being anywhere between one day and three years.

Let $P(E) = q$. Now

$$P(E|X=x) = P(X=x|E)\frac{P(E)}{P(X=x)} = \frac{cq}{cq+(1-q)f(x)} \tag{5}$$

Given estimates of q and $f(\cdot)$, to find t_0 we may set this expression to $1/2$ and solve for x.

In order to estimate q and f with $\hat{q}$ and $\hat{f}$, we consider the population P of admissions (not individuals) where the admission date is between May 2013 and May 2014. We then estimate

$$\begin{aligned}\hat{q} &= \text{proportion of } P \text{ with no recorded discharge date or discharged after May 2016}\\ \hat{f}(x) &= \text{proportion of } P \text{ with recorded discharge date before May 2016 with length of stay } x\end{aligned}$$

We use this population of admissions so as to avoid data leakage, since these are prior to the earliest time cutoff (May 2016) used in fitting the model. This is also our rationale for treating individuals who were discharged post-May 2016 the same as having no recorded discharge date: we cannot use this information without data leakage. However, we note that the number of individuals with genuine >2 year hospital stays is very small.

Following this procedure, estimated values of t_0 are 26, 19 and 6 for `emergency_bed_days`, `elective_bed_days` and `other_bed_days`, respectively.

5 Assessment of calibration

We use an estimator for calibration broadly based on the Nadaraya-Watson kernel estimator [7, 8]. We re-derive several properties (consistency, bias) to highlight their interpretation in our context.

We assume in general that, for IID predictor/outcome pairs $(X_i, Y_i) \sim (X, Y)$, $i \in 1..n$, and an optimal predictor function p_{opt}, we have

$$Y|X \sim \text{Bernoulli}[p_{opt}(X)] \tag{6}$$

noting that this implies

$$p_{opt}(X) = E[Y|p_{opt}(X)] \tag{7}$$

We want to estimate $p_{opt}(X)$.

Since we only observe $Y=1$ or $Y=0$, we must estimate $E[Y|p(X)=z]$ as some kind of average of Y about observed values $p(X)$ close to z. A routine way to do this is to use 'reliability diagrams' [9] in which we bin values of $p(X)$ and estimate $E(Y|p(X))$ in each bin.

Since for small bin sizes there may be few or no values of $p(X)$ in some bins, we use a kernel estimate $\hat{c_p}(z)$ of $c_p(z) = E[Y|p(X)=z]$:

$$\hat{c_p}(z) = z\frac{\sum_i Y_i K_\delta[p(X_i), z)]}{\sum_i p(X_i)K_\delta[p(X_i), z]} = \sum_i w_i Y_i \tag{8}$$

where $K_\delta : (0,1)^2 \to \mathbb{R}^+$ is some distance-measuring kernel with width δ, and

$$w_i = z\frac{K_\delta[p(X_i), z)]}{\sum_i p(X_i)K_\delta[p(X_i), z]} \tag{9}$$

We avoid the simpler estimate given by the K_δ-weighted mean of Y_is:

$$\tilde{c_p}(z) = \frac{\sum_i Y_i K_\delta[p(X_i), z)]}{\sum_i K_\delta[p(X_i), z]} \tag{10}$$

for reasons shown below. We note the following:

Proposition 1. *If $p(X)$ has Lesbegue-integrable positive density on $(0,1)$, $K(z,x)$ and $c_p(x)$ are Lesbegue-integrable functions of x for fixed $z>0$, and the kernel 'narrows with δ' so*

$$\begin{aligned}E_X\{p(X)K_\delta[p(X), z]\} &\xrightarrow{\delta\to 0} z\\ E_X\{c_p[p(X)]K_\delta[p(X), z]\} &\xrightarrow{\delta\to 0} c_p(z)\end{aligned}$$

then $\hat{c}(z)$ becomes a consistent estimator of $c(z)$ as $\delta \to 0$

Proof. From Slutsky's lemma, the law of total expectation and the strong law of large numbers

$$\hat{c_p}(z) = z\frac{\sum_i Y_i K_\delta(p(X_i), z)}{\sum_i f(X_i) K_\delta(f(X_i), z)} \xrightarrow[\text{prob}]{n\to\infty} z\frac{E_X\left\{c_p[p(X)]K_\delta[f(X), z]\right\}}{E_X\left\{p(X)K_\delta[p(X), z]\right\}} \xrightarrow{\delta\to 0} z\frac{c_p(z)}{z} = c_p(z) \tag{11}$$

□

We note that $\hat{c_p}(Z)$ is not generally consistent if $\delta > 0$. However, the inconsistency is not severe: we note

Proposition 2. *If, in addition to the above, $K_\delta(x,z) = K_\delta(x-z)$ is a symmetric density with second moment δ and negligible moments of higher order, and the densities of $p(X)$ and $c_p(X)$ are twice differentiable at z, then $\hat{c_p}(z) \to c_p(z) + O(\delta^2)$*

Proof. We have

$$\begin{aligned}
E_X\{c_p[p(X)]K_\delta[p(X), z]\} &= E_{x\sim p(X)}[c_p(x)K_\delta(x-z)] \\
&= \int_0^1 f_{p(X)}(x)c_p(x)K_\delta(x-z)dx \\
&= \int_0^1 (f_{p(X)}(z) + f'_{p(X)}(z)(x-z))(c_p(z) + c'_p(x-z))K_\delta(x-z)dx \\
&\quad + \int_0^1 O\left((x-z)^2\right)K_\delta(x-z)dx \\
&= f_{p(X)}(z)c_p(z) + \int_0^1 O\left((x-z)^2\right)K_\delta(x-z)dx \\
&\quad + (f_{p(X)}(z)c'_p(z) + f'_{p(X)}(z)c_p(z))\int_0^1 (x-z)K_\delta(x-z)dz \\
&= f_{p(X)}(z)c_p(z) + O\left(\delta^2\right)
\end{aligned} \tag{12}$$

noting the symmetry of K_δ. If we replace $c_p[p(X)]$ with $p(X)$, the expectation is $zf_{p(X)}(z) + O(\delta^2)$, and the result follows from the first part of 11. □

Remark 1. *In the ideal case where $c_p(z) = z$ (that is, our model is perfectly calibrated) estimator 9 is consistent even when $\delta > 0$, whereas the apparently simpler asymptotically consistent (as $\delta \to 0$) estimator of a weighted sum of Y_i's:*

$$\tilde{c_p}(z) = \frac{\sum_i Y_i K_\delta[p(X_i), z)]}{\sum_i K_\delta[p(X_i), z]} \tag{13}$$

is not.

Finally, we note the following:

Proposition 3. *Under the assumptions above, with fixed X_i, the bias of $\hat{c_p}(z)$ is*

$$\frac{\sum_i B(X_i, z)K_\delta[p(X_i), z])}{\sum_i p(X_i)K_\delta[p(X_i), z]} = \sum_i w_i \frac{B(X_i, z)}{z} \tag{14}$$

where $B(X_i, z) = p(X_i)c_p(z) - zc_p(p(X_i))$.

Proof. With fixed X_i

$$\begin{aligned}
E_Y[c_p(z) - \hat{c_p}(z)] &= E_Y\left[c_p(z) - \sum_i w_i Y_i\right] \\
&= c_p(z) - z\frac{\sum_i c_p(p(X_i))K_\delta[p(X_i), z)]}{\sum_i p(X_i)K_\delta[p(X_i), z]} \\
&= \frac{\sum_i [p(X_i)c_p(z) - zc_p(p(X_i))]K_\delta[p(X_i), z])}{\sum_i p(X_i)K_\delta[p(X_i), z]} \\
&\sum_i w_i \frac{B(X_i, z)}{z}
\end{aligned}$$

as required. □

Remark 2. *This enables straightforward evaluation of bounds on bias given bounds on the form of c_p. The estimator $\hat{c_p}$ is unbiased if $c_p(x) = kx$ for some k, since $B(X_i, z) \equiv 0$.*

Remark 3. *An alternative way to draw a kernelised calibration curve is to simply plot a parametric curve*

$$\begin{pmatrix} x(t) \\ y(t) \end{pmatrix} = \begin{pmatrix} \sum p(X_i) K_\delta[p(X_i), t] \\ \sum Y_i K_\delta[p(X_i), t] \end{pmatrix} \tag{15}$$

which, for each t, is an only-slightly biased estimate of some point $z, c_p(z)$. If a rectangular kernel is used, this is equivalent to binning values of $p(X_i)$ [9]. However, this method does not generally give a curve across the entire range of $p(X_i)$.

It is straightforward to estimate

$$\begin{aligned}
\text{var}(c_p(z) \mid \{X_1, X_2 \ldots X_n\}) &= \text{var}\left(z \frac{\sum_i Y_i K_\delta[p(X_i), z]}{(\sum_i p(X_i) K_\delta[p(X_i), z])} \,\middle|\, X_1, X_2 .. X_n \right) \\
&= \text{var}(\sum_i w_i Y_i \mid \{X_1, X_2 .. X_n\}) \\
&= \sum_i w_i^2 \text{var}(Y_i \mid X_1, X_2, .. X_n) \\
&\approx \sum_i w_i^2 p(X_i)(1 - p(X_i))
\end{aligned}$$

where the approximation is exact if $c_p(z) = z$. Together with an estimate of maximum absolute bias b_z at z, this enables estimates of conservative confidence intervals on $\hat{c_p}(z)$ at level $1 - \alpha$:

$$\hat{c_p}(z) \pm \left(b_z + \Phi^{-1} \left(\frac{\alpha}{2} \right) \text{SE}(c_p(z) | X_i) \right) \tag{16}$$

In all plots in this paper, we bounded bias under the assumption that there existed k such that $|c_p(z) - kz| < z^2/10$.

The calibration estimator derived here is demonstrated in an R script `sparra_calibration.R` available with the attached R code for this manuscript.

SUPPLEMENTARY REFERENCES

[1] Gary S Collins, Johannes B Reitsma, Douglas G Altman, and Karel GM Moons. Transparent reporting of a multivariable prediction model for individual prognosis or diagnosis (tripod): the tripod statement. *Journal of British Surgery*, 102(3):148–158, 2015.

[2] Karel GM Moons, Douglas G Altman, Johannes B Reitsma, John PA Ioannidis, Petra Macaskill, Ewout W Steyerberg, Andrew J Vickers, David F Ransohoff, and Gary S Collins. Transparent reporting of a multivariable prediction model for individual prognosis or diagnosis (TRIPOD): explanation and elaboration. *Annals of internal medicine*, 162(1):W1–W73, 2015.

[3] Public Health Scotland. SMR datasets - ISD Scotland Data Dictionary, 2023. `https://www.ndc.scot.nhs.uk/Data-Dictionary/SMR-Datasets/`, Accessed: 6-3-2023.

[4] Public Health Scotland. AE2 - Accident and emergency records, 2020. `hhttps://www.ndc.scot.nhs.uk/National-Datasets/data.asp?SubID=3`, Accessed: 6-3-2020.

[5] Public Health Scotland. System Watch: urgent care usage, 2020. `https://publichealthscotland.scot/services/system-watch/#section-1-1`, Accessed: 6-3-2023.

[6] Public Health Scotland. PIS - Prescribing information systems, 2020. `https://www.ndc.scot.nhs.uk/National-Datasets/data.asp?SubID=9`, Accessed: 6-3-2020.

[7] Elizbar A Nadaraya. On estimating regression. *Theory of Probability & Its Applications*, 9(1):141–142, 1964.

[8] Geoffrey S Watson. Smooth regression analysis. *Sankhyā: The Indian Journal of Statistics, Series A*, pages 359–372, 1964.

[9] Jochen Bröcker and Leonard A Smith. Increasing the reliability of reliability diagrams. *Weather and forecasting*, 22(3):651–661, 2007.
